# Eggplant Resistance to the *Ralstonia solanacearum* Species Complex Involves Both Broad-Spectrum and Strain-Specific Quantitative Trait Loci

**DOI:** 10.3389/fpls.2017.00828

**Published:** 2017-05-19

**Authors:** Sylvia Salgon, Cyril Jourda, Christopher Sauvage, Marie-Christine Daunay, Bernard Reynaud, Emmanuel Wicker, Jacques Dintinger

**Affiliations:** ^1^UMR Peuplements Végétaux et Bioagresseurs en Milieu Tropical, Centre de Coopération Internationale en Recherche Agronomique pour le DéveloppementSaint-Pierre, Réunion; ^2^Association Réunionnaise pour la Modernisation de l’Economie Fruitière, Légumière et HORticoleSaint-Pierre, Réunion; ^3^UMR Peuplements Végétaux et Bioagresseurs en Milieu Tropical, Université de la RéunionSaint-Pierre, Réunion; ^4^UR 1052 Génétique et Amélioration des Fruits et Légumes, Institut National de la Recherche AgronomiqueMontfavet, France; ^5^UMR Interactions Plantes-Microorganismes-Environnement, Centre de Coopération Internationale en Recherche Agronomique pour le DéveloppementMontpellier, France

**Keywords:** plant–pathogen interaction, *Solanum melongena*, bacterial wilt, quantitative resistance, candidate genes

## Abstract

Bacterial wilt (BW) is a major disease of solanaceous crops caused by the *Ralstonia solanacearum* species complex (RSSC). Strains are grouped into five phylotypes (I, IIA, IIB, III, and IV). Varietal resistance is the most sustainable strategy for managing BW. Nevertheless, breeding to improve cultivar resistance has been limited by the pathogen’s extensive genetic diversity. Identifying the genetic bases of specific and non-specific resistance is a prerequisite to breed improvement. A major gene (*ERs1*) was previously mapped in eggplant (*Solanum melongena* L.) using an intraspecific population of recombinant inbred lines derived from the cross of susceptible MM738 (S) × resistant AG91-25 (R). *ERs1* was originally found to control three strains from phylotype I, while being totally ineffective against a virulent strain from the same phylotype. We tested this population against four additional RSSC strains, representing phylotypes I, IIA, IIB, and III in order to clarify the action spectrum of *ERs1*. We recorded wilting symptoms and bacterial stem colonization under controlled artificial inoculation. We constructed a high-density genetic map of the population using single nucleotide polymorphisms (SNPs) developed from genotyping-by-sequencing and added 168 molecular markers [amplified fragment length polymorphisms (AFLPs), simple sequence repeats (SSRs), and sequence-related amplified polymorphisms (SRAPs)] developed previously. The new linkage map based on a total of 1,035 markers was anchored on eggplant, tomato, and potato genomes. Quantitative trait locus (QTL) mapping for resistance against a total of eight RSSC strains resulted in the detection of one major phylotype-specific QTL and two broad-spectrum QTLs. The major QTL, which specifically controls three phylotype I strains, was located at the bottom of chromosome 9 and corresponded to the previously identified major gene *ERs1*. Five candidate R-genes were underlying this QTL, with different alleles between the parents. The two other QTLs detected on chromosomes 2 and 5 were found to be associated with partial resistance to strains of phylotypes I, IIA, III and strains of phylotypes IIA and III, respectively. Markers closely linked to these three QTLs will be crucial for breeding eggplant with broad-spectrum resistance to BW. Furthermore, our study provides an important contribution to the molecular characterization of *ERs1*, which was initially considered to be a major resistance gene.

## Introduction

*Ralstonia solanacearum*, a widespread soil-borne pathogen present in all continents, is the causal agent of bacterial wilt (BW) disease. The pathogen enters the plant via the roots, then colonizes the xylem vessels and spreads through the vascular system of susceptible plants. It causes typical wilting symptoms, leading to the host plant’s rapid death. BW has been reported on cash crops, such as tobacco, as well as on major food crops, such as banana, potato, tomato, and eggplant. Recently, it has been ranked second in the list of the most scientifically/economically important bacterial pathogens (Mansfield et al., 2012). *R. solanacearum* was considered as a species complex for the first time by Gillings and Fahy (1994) because it includes a large number of genetic groups. Later, the *R. solanacearum* species complex (RSSC) was subdivided into five monophyletic groups called phylotypes. A probable geographical origin has been attributed to each phylotype: phylotype I strains originate from Asia, phylotype IIA strains from the north of Latin America and the Caribbean, phylotype IIB strains from South America, phylotype III strains from Africa, and phylotype IV strains from Indonesia, Australia, and Japan (Fegan and Prior, 2005; Wicker et al., 2012). Using both a comparison of sequenced genomes and a polyphasic classical taxonomy approach, RSSC was recently subdivided into three genomic species: (i) *Ralstonia solanacearum*, including phylotype IIA and IIB; (ii) *R. pseudosolanacearum*, including phylotypes I and III; and (iii) *R. syzygii*, including the former *R. solanacearum* phylotype IV and the clove pathogen *R. syzygii* (Safni et al., 2014). Although a recent study combining genomics, proteomics and functional phenotypic assays confirmed this classification (Prior et al., 2016), its ecological and evolutionary relevance is not yet known.

*Ralstonia solanacearum* species complex strains represent a significant threat for crops because of their unusually broad range of host plants (more than 200 plant species), their extensive genetic diversity and persistence in the field (Genin and Denny, 2012). Strategies to manage BW, such as crop rotation, elimination of weeds that provide alternative hosts and biological control are insufficient and the disease still causes major profit loss (Huet, 2014). Thus, breeding resistant cultivars that have broad spectrum-resistance to diverse strains of RSSC is an important part of a composite strategy for controlling BW in infested areas. Until now, the genetic mechanisms underlying resistance have been studied almost exclusively in model plant species. In *Arabidopsis thaliana*, several molecular studies led to the identification of the major resistance gene *RRS1*, coding for a TIR-NBS-LRR resistance protein, which interacts directly with the avirulence effector PopP2 (Deslandes et al., 1998, 2002, 2003; Lahaye, 2004). *RRS1* also requires the presence of a Cys protease, encoded by the *RD19* gene, to mediate resistance to GMI1000, a phylotype I strain (Bernoux et al., 2008). More recently, the *RRS1* gene was found to work closely with the *RPS4* gene (resistance to *Pseudomonas syringae 4*), increasing resistance to both *Pseudomonas* and RSSC strains with AvrRps4 and PopP2 effectors, respectively (Narusaka et al., 2009; Sohn et al., 2014). *A. thaliana* quantitative BW-resistance is mediated by the *ERECTA* gene, a member of the receptor-like kinase (RLK) family (Godiard et al., 2003). The latest gene identified, which was found to be associated with *A. thaliana* resistance to BW, is *wat1* (walls are thin1). It confers broad-spectrum resistance to vascular pathogens, including RSSC strains, *Xanthomonas campestris* and *Verticillium dahliae* (Denancé et al., 2013). BW-resistance studies have also been carried out on crops, mainly on solanaceous species. However, these studies focus on resistance inheritance and rarely characterize the molecular mechanisms. In the tomato (*S. lycopersicum*) accession Hawaii 7996, BW-resistance is controlled by two major and several minor quantitative trait locus (QTLs). The major QTLs called *bwr-12* and *bwr-6*, confer partial resistance to phylotype I and both phylotypes I and IIB strains, respectively (Wang et al., 2000, 2013; Carmeille et al., 2006). In eggplant (*Solanum melongena L*.), a major gene (*RE-bw*) was cloned. The RE-bw protein was found to interact with the type III effector PopP2 (Xi’ou et al., 2014). Furthermore, wild relatives of eggplant have been shown to harbor highly effective resistance factors, especially in the *S. aethiopicum* Aculeatum group (generally referred to as *S. integrifolium*) and *Solanum torvum*, which exhibits HR in the presence of the type III effector Rip36 (=RipAX2) (Nahar et al., 2014). A panel of 10 eggplant accessions, tested under controlled conditions against a core collection of 12 strains representative of all RSSC phylotypes (Lebeau et al., 2010), revealed that eggplant is a potential host for all phylotypes. It also exhibits a wide range of incompatible reactions (absence of wilting symptoms and colonization). These results indicate that eggplant and some related *Solanum* species probably harbor major strain-specific BW-resistance genes.

Eggplant and its close wild relatives also display resistance for a broad range of pathogens (Daunay, 2008; Syfert et al., 2016). In cultivated eggplant, introgression breeding of resistance, originating from related species, has been limited until now (Daunay, 2008). However, crossability studies have illustrated the potential of introgression breeding (Behera and Singh, 2002; Premabati et al., 2015; Kaushik et al., 2016; Plazas et al., 2016). Whereas the majority of Solanaceae crop species originate from the New World (tomato, potato, and pepper), eggplant and its wild relatives are native to the Old World. Eggplant’s ancestor was East African and probably spread to Asia via the Middle East, either spontaneously or during human migrations (Weese and Bohs, 2010). Eggplant seems to have been domesticated in Southeast Asia. The long-lasting co-evolution between eggplant and its Old World pathogens may explain why specific resistance genes do not exist in solanaceous crops in the New World (Hirakawa et al., 2014). Similar to tomato and pepper, eggplant is an autogamous diploid species with 12 chromosomes and a genome size estimated at 1.1 Gb (Arumuganathan and Earle, 1991). Despite its agronomical importance, eggplant genetics and genomics are less documented than other major solanaceous crops. Given the absence of high-density linkage maps for eggplant, it is difficult to detect reliable QTLs in short delimited genomic intervals and to identify the underlying putative resistance alleles. Thanks to the recent publication of the first sequenced draft genome of eggplant (Hirakawa et al., 2014) and the decreasing cost of high-throughput sequencing technologies (HTS), it is now easier to construct high-resolution maps for this species. Among the HTS methods, the genotyping-by-sequencing (GBS) method has been widely used to genotype thousands of individuals at a relatively low cost, by multiplexing samples with individual barcodes (Elshire et al., 2011). In eggplant, an SNP-based linkage map inferred from GBS would facilitate comparison between genetic and physical maps of syntenic crops, in particular tomato and potato, which have been studied extensively. Thus, with the recent advances in marker discovery and eggplant’s special position in Solanaceae phylogeny, eggplant constitutes an outstanding model for mapping and comparing resistance factors to pathogens and their underlying molecular mechanisms.

Eggplant is potentially resistant to all the RSSC phylotypes. Therefore, it is a particularly interesting species for the study of BW-resistance. In this context, a population of recombinant inbred lines (RILs), from a cross between a susceptible parent (line MM738) and a resistant parent (line AG91-25), was phenotyped with phylotype I strains by Lebeau et al. (2013). An intraspecific map of the population was set up with 119 molecular markers positioned on 18 linkage groups. This study led to the detection of a major monogenic resistance locus (called *ERs1*), a unique case in crop RSSC resistance. However, the low density of this map, the lack of anchor markers, together with the very low level of molecular polymorphism in the RIL population, limited further investigation into the genetics of AG91-25 RSSC resistance. Our primary goal was to develop tools for breeding broad-spectrum resistant cultivars. Therefore, we focused on the genetic architecture of AG91-25 resistance, with the following objectives:

(i) Determine the phylotype spectrum of *ERs1*-associated resistance to a collection of RSSC strains encompassing all phylotypes that affect solanaceous crops (phylotypes I, IIA, IIB, and III).
(ii) Identify other QTLs contributing either to broad host resistance or phylotype-specific resistance.
(iii) Determine physical position of *ERs1* and other identified QTLs by constructing a dense anchored genetic map; investigate whether the resistance loci contain orthologous genes of already known R-genes, by using the tomato reference genome as a comparison.

## Materials and Methods

### Plant Material and RSSC Strains

We studied a population of 180 RILs, derived from the cross between *S. melongena* MM738 (susceptible) and *S. melongena* AG91-25 (resistant to BW). This population, previously phenotyped for BW-resistance to four strains of phylotype I (Lebeau et al., 2013), was exposed to four new strains belonging to phylotypes I, III (*R. pseudosolanacearum* sensu lato, Safni et al., 2014), IIA and IIB (*R. solanacearum* sensu lato, Safni et al., 2014). **Table 1** provides further information on the eight strains, in particular their geographical origin and the host on which they were isolated. All strains are very aggressive on the susceptible parent and display different levels of aggressiveness on the resistant parent (Lebeau et al., 2010). We chose not to consider resistance to phylotype IV strains (*R. syzygii* subsp. *indonesiensis* sensu lato, Safni et al., 2014) because of the quarantine restriction in Réunion and also because this phylotype was reported to be of low epidemiological potential (Wicker et al., 2012) and of little agronomic importance for Solanaceae (Lebeau et al., 2010).

**Table 1:** Description of the eight *R. solanacearum* species complex strains used for phenotyping the eggplant [MM738 × AG91-25] RIL population.

Strain	Alternative name	Host of origin	Country	Classification^a^
GMI1000^b^	RUN0054, JS753	*Solanum lycopersicum*	French Guyana	I-18
PSS366^b^	RUN0155	*Solanum lycopersicum*	Taiwan	I-15
CMR134^b^	RUN0215, CFBP7058	*Solanum scabrum*	Cameroon	I-13
PSS4^b^	RUN0157, CIP410	*Solanum lycopersicum*	Taiwan	I-15
TO10^c^	RUN0969	*Solanum lycopersicum*	Thailand	I-47
CFBP2957^c^	RUN0036, MT5	*Solanum lycopersicum*	Martinique	IIA-36
CFBP3059^c^	RUN0039, JS904	*Solanum melongena*	Burkina Faso	III-23
CMR34^c^	RUN0147, CFBP7029	*Solanum lycopersicum*	Cameroon	IIB-1

*^a^Classification using the Phylotype–Sequevar nomenclature (Fegan and Prior, 2005). ^b^Strains used for RIL population phenotyping in Lebeau et al. (2013). ^c^Strains used for RIL population phenotyping in the present study.*

### DNA Extraction, Library Construction, and Sequencing

DNA was extracted from a bulk of young leaves using a modified CTAB procedure (Doyle and Doyle, 1987). The quality and quantity of DNA were analyzed using the Qubit fluorometric technology (Thermo Fisher Scientific, Illkirch, France) and agarose gel electrophoresis. GBS libraries were produced by the Global Genetik Teknolojileri (GGT) Company (Istanbul, Turkey) according to the protocol developed by Elshire et al. (2011) and using barcode sequences (for details, see Supplementary Table S1). Libraries were sequenced using Illumina technology with DNA-seq paired-end protocols on a HiSeq2000 sequencer (Illumina, Inc., San Diego, CA, United States) by the Wisconsin Biotech Center.

### Sequence Data Processing and Polymorphism Detection

Illumina adapter sequences were removed from raw reads and remaining reads were assigned to individual lines using barcode sequences (one specific barcode sequence for each line) with the GBS barcode splitter tool^1^. Barcode sequences were trimmed, unassigned reads were removed and remaining reads were trimmed to 90 bp. The STACKS software v.1.28 (Catchen et al., 2013) was used to identify single nucleotide polymorphisms (SNPs) in the 180 RIL population and their two parental lines. First, reads of low quality (below an average phred score of 10 with a sliding window of 15 bp) and reads with uncalled bases were removed using the process_radtags program. Remaining reads were merged into one single file per individual line using a concatenate function. The quality of cleaned reads was checked using the FastQC tool (Andrews, 2010). Next, cleaned reads were assembled and genotyped using both the *de novo* pipeline (without the use of a reference genome) and the *reference-guided* pipeline (after mapping reads on the available eggplant genome) in STACKS.

For the reference-guided pipeline, bowtie software version 1.1.1 (Langmead et al., 2009) was used to map cleaned reads on the *S. melongena* genome SME_r2.5.1 (Hirakawa et al., 2014). Only the best uniquely mapped reads with a maximum of one mismatch were selected. For the two methods, three identical reads were required to produce a locus. The catalog of loci was constructed from six samples including parental lines MM738 and AG91-25 and four individuals of the progeny with highest sequencing depths. Additional filters were added to select significant SNPs:

– Loci present in at least 40% of individuals;
– Minimum SNP allele frequency of 5% in the RIL population;
– Maximum SNP heterozygosity of 15% in the RIL population.

The minimum SNP allele frequency was set to the low value of 5% according to the study conducted by Lebeau et al. (2013) in which BW-resistance was associated to highly distorted markers. We allowed the high heterozygosity rate of 15% in RILs because of genotyping errors and bias in SNPs, where up to 60% of data was missing. Erroneous loci containing paralogous sequences or repetitive sequences were expected to contain more polymorphisms than true loci with different allelic sequences only (Tang et al., 2006; Treangen and Salzberg, 2012). Thus, highly polymorphic loci (>3%) were discarded to avoid potentially false positive SNPs. Missing genotyping data were imputed using a classification method called “random forest” (Breiman, 2001; Poland et al., 2012) implemented in the missForest package (Stekhoven and Bühlmann, 2012). The package also provided the proportion of falsely classified entries (PFC) for imputed data.

### Genetic Map Construction

The genetic map was constructed from all SNPs that were polymorphic between the two parents of the RIL population, using JoinMap^®^ 4.1 (Van Ooijen, 2006). A minimum independence value of logarithm of odds score (LOD) of 5.0 was attributed in order to group the markers. Linkage groups containing less than three markers were discarded. Resulting linkage groups were merged when markers from one group indicated cross-links (SCL) with markers from another group. Recombination fractions were converted to map distances in centimorgans (cM) using the Kosambi mapping function. Markers with a recombination frequency > 0.7 and LOD > 1 were declared unrelated to a linkage group and consequently removed. Perfectly identical markers (similarity value of 1) were excluded before ordering markers to speed up the analysis time. Markers were ordered using the default values of JoinMap. After ordering, the mean chi-square contributions of each marker were computed. A high chi-square contribution indicates that the marker does not display a good fit in the linkage group. Thus, markers with mean-square contributions over five were removed and the remaining markers were re-ordered. In order to compare our map with published Solanaceae maps, catalogs of our loci were aligned on the eggplant (*Solanum melongena*) genome (SME_r2.5.1; Hirakawa et al., 2014), the tomato (*Solanum lycopersicum*) genome (SL2.50; Sato et al., 2012) and the potato (*Solanum tuberosum*) genome (PGSC_DM_v4.03; Xu et al., 2011) using the Blastn program in the NCBI’s BLAST+ v 2.2.28 software (Camacho et al., 2009). The cut-off values for a significant hit were fixed at 1 × 10^-20^ for the eggplant genome and 1 × 10^-15^ for tomato and potato genomes. Maps were plotted using MapChart 2.2 (Voorrips, 2002).

### Resistance Assays and Phenotypic Data Analysis

#### Experimental Design

We performed resistance assays for the four new RSSC strains on a set of 158 to 180 F_6-7_ RILs at the experimental station of the Centre de Coopération Internationale en Recherche Agronomique pour le Développement (CIRAD) in Saint-Pierre, Réunion Island (140 m elevation, 21°S, 55.3°E). The number of phenotyped RILs differed slightly between assays, depending on their germination rate and post-transplanting mortality rate. An additional strain, TO10 (**Table 1**) was tested at CIRAD (assay nickname “TO10.Réunion”) and at an experimental station belonging to a partner seed company in central Java, Indonesia (339 m elevation, -8°N, 110°E) (assay nickname “TO10.Indonesia”). Each assay was conducted in two greenhouses during two seasons between 2010 and 2015, representing four replicates, following protocols described below. Each replicate included five plants of each RIL family, 85 to 125 plants of each parent, 20 plants of the F_1_ and 100 plants of each BC_1_ generation. Plants were arranged using a randomized complete block design to control environmental effects. For “TO10.Indonesia” assay, two replications of eight plants for each RIL family and 12 plants of each parental line were implemented. Supplementary Table S2 shows the details for each assay, including environmental conditions (period, temperature, and relative humidity).

#### Inoculation and Disease Assessment

*Ralstonia solanacearum* species complex strains were grown at 28°C on 2,3,5-triphenyl tetrazolium chloride medium (Kelman, 1954). Actively growing cultures were harvested after a 24 h incubation period, by flooding plates with 5 mL of Tris buffer (Trizma 0.01 M pH 7.2: Sigma, St Louis, MO, United States). The bacterial suspension was then titrated by spectrophotometry to adjust the inoculum concentration at approximately 1 × 10^8^ CFU per mL (OD_600nm_ = 0.1).

Plants were inoculated at the 4–5 fully expanded leaf stage, after a preliminary knife-scarification of the roots. The bacterial suspension was drenched through the drip irrigation system immediately after the roots were damaged with a knife. In this way, each plant was inoculated with an estimated average of 100–200 mL at 10^6^ CFU per mL. For tests in Indonesia, each plant was inoculated with 30 mL of a bacterial suspension at 10^8^ CFU per mL.

Symptom scoring was carried out twice a week using a wilting scale, as defined by Lebeau et al. (2013). For every scoring date, we computed the wilt mean score (mean rating of all plants from each replicate, coded SCO), as well as the proportion of wilted plants (proportion of plants with a score ≥ 1, coded W) for each recombinant line, the parents and the progenies. At the end of the assay, the presence of latent infection was tested on all asymptomatic plants: a section of each plant stem base was sampled, transferred into a tube filled with 5 mL Tris buffer and left for 1–2 h at room temperature to allow bacteria to stream out of the xylem vessels. Thereafter, 50 μl was streaked onto a plate of selective medium (Granada and Sequeira, 1983) and incubated at 28°C for 3 days. A plate was considered positive when RSSC colonies were visible. The Colonization Index (CI) of each RIL was then computed (Lebeau et al., 2013). The area under the disease progress curve (AUDPC) was computed at the end of each assay for (i) the score (SCOaudpc) and (ii) the percentage of wilted plants (Waudpc). For the test in Indonesia, scoring was restricted to the incidence of BW (number of wilted plants in each family) and carried out once a week for 4 weeks. The W value was calculated at each scoring date and the AUDPC value was computed for the proportion of wilted plants at the end of the assay.

### Statistical Analysis of Phenotypic Data and QTL Mapping

Descriptive statistics and analysis of variance (ANOVA) were carried out using R software (R Core Team, 2014). Phenotypic variables W, SCO, CI, Waudpc, and SCOaudpc were computed, as described previously (Carmeille et al., 2006; Lebeau et al., 2013). Phenotypic correlations between variables were estimated using the Pearson coefficient for each assay. ANOVA on Waudpc was computed for each strain using the linear model implemented in the R package stats. The ANOVA fixed effects model for each individual assay took into account only the “genotype” and “greenhouse” factors. The ANOVA fixed effects model for combined seasons took into account the “genotype,” “season,” and “greenhouse nested within the season” factors, as well as the interaction between “genotype” and “season.” Genotypic and environmental variance was calculated using ANOVA results to estimate the broad-sense heritability (*h*^2^) (Hallauer and Miranda, 1981). The normal distribution of phenotype values was tested with Shapiro–Wilk tests (normality was accepted for *P*-values > 0.05). Phenotypic data from assays with strains GMI1000, PSS366, and CMR134 were analyzed separately. They were also combined, insofar as the strain effect in the ANOVA model was not found to be significant by Lebeau et al. (2013). For the purposes of consistency with previous work, data from strains GMI1000, PSS366, and CMR134 were considered together and named “grouping,” whereas data from individual strains analyzed across two seasons were named “combined.” According to their W and CI values, parental lines and progeny were attributed to the six phenotypic classes defined in Lebeau et al. (2010): 1 = highly resistant, 2 = moderately resistant, 3.1 = partially resistant, 3.2 = latent infection, 4 = moderately susceptible, 5 = highly susceptible.

QTL mapping was performed for Waudpc variable using the R/qtl package version 1.39 (Broman et al., 2003). R/qtl considers a RIL population as fixed (without heterozygote genotypes) and treats the heterozygotes as missing data. An initial scan was performed using Simple Interval Mapping (SIM) (Lander and Botstein, 1989) with a 1 cM step and a non-parametric model. All variables with a distribution close to normality were then analyzed with Composite Interval Mapping (CIM) (Zeng, 1994) by using a parametric model with the simple regression method (Haley and Knott, 1992). The number of covariates was fixed according to the number of QTLs detected in SIM analysis. Another model with the detected QTLs was created and their positions were re-estimated using the makeqtl command and the refineqtl command with default parameters, respectively. Lastly, the fitqtl function was used to estimate the proportion of phenotypic variation explained by each QTL (*R*^2^), as well as the additive effects of each QTL. The digenic epistatic interaction between QTLs pairs was also tested. The Waudpc means were compared for “AA” and “BB” genotypes (corresponding to the genotype of the susceptible parent and the genotype of the resistant parent, respectively) at each QTL, using a Fisher’s least-significance-difference test (LSD) (Hayter, 1986). In order to detect potential closely linked QTL, an automated stepwise model selection, scanning for additive and epistatic QTL, was performed using the stepwiseqtl function (Manichaikul et al., 2009).

Phenotypic variables obtained in assays with strains GMI1000, PSS366, and CMR134, displaying strong deviations from normality, were also analyzed using a binary model and the Haley Knott (HK) regression. For the binary model, Waudpc was coded as “0” when the value was ≤10 and “1” elsewhere. These values were chosen because they clearly differentiate two classes from the bimodal distributions of the phenotypes. The LOD thresholds, which indicate “significant” for any QTLs, were estimated using 1,000 permutations with a genome-wide significance level of α = 0.05 (Churchill and Doerge, 1994) for the normal and binary models, and α = 0.01 for the non-parametric model. The Haley Knot regression was used for its speed and robustness against non-normality (Rebaï, 1997). The confidence interval for each QTL peak was derived from the Bayesian 95% credible interval using the bayesint function implemented in R/qtl. All the QTLs detected were named as follows: EBWR ‘linkage group number’ (EBWR, the acronym for Eggplant Bacterial Wilt Resistance).

### Searching for Candidate R-Genes Underlying the Detected QTLs

The ITAG2.4 tomato annotated transcriptome (Sato et al., 2012^2^) and the annotated transcriptome of the susceptible parental line MM738 (Sarah et al., 2016^3^) were used to search typical R-genes belonging to the TNL (TIR-NB-LRR), the CNL (CC-NB-LRR), the RLP (receptor-like proteins) and the RLK classes. Transcripts of the MM738 accession were mapped on the eggplant draft genome using the “Exonerate version 2.2.0” software with the est2genome model (Slater and Birney, 2005). RNA-seq reads of the resistant eggplant AG91-25 were produced, cleaned and assembled following the method published previously (Sarah et al., 2016). Cleaned reads of the two parents were mapped onto the MM738 transcriptome using the Burrows–Wheeler Aligner (Li and Durbin, 2009) and the variant calling was performed using the Genome Analysis Toolkit, version 3.3 (McKenna et al., 2010). Candidate R-genes found in QTL regions were further analyzed by comparing the parental line alleles. We also searched candidate R-genes in transcripts which are uniquely present in AG91-25 line. Sequences of cloned BW-resistance genes *RRS1* and *RPS4* (genes ID 834562 and 834561; Deslandes et al., 1998, 2003), *RD19* (gene ID 830064; Bernoux et al., 2008), *RE-bw* (Accession ID JQ429763.1; Xi’ou et al., 2014), *ERECTA* (gene ID 817173; Godiard et al., 2003) and *wat1* (gene ID 843886; Denancé et al., 2013) were aligned on tomato and eggplant genomes using the BLAST tool included on the SOL genomics network website^4^.

## Results

### A New Dense Genetic Map of Eggplant, Anchored on Tomato, Potato, and Eggplant Genomes

**FIGURE 1:**
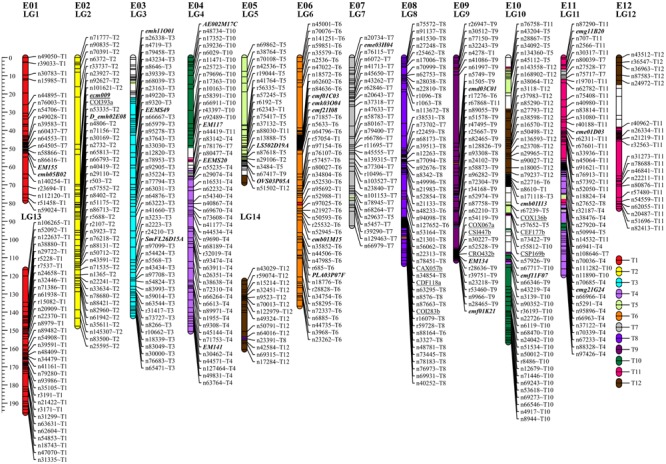
**Genetic map of eggplant [MM738 × AG91-25] RIL population anchored on eggplant and tomato chromosomes.** Tomato chromosomes syntenic to eggplant chromosomes are indicated by the suffix “Tx” at the end of each marker name. They are illustrated with a color code (key at the bottom right of the figure). SSR markers are indicated in bold italics; SNPs from the *de novo* pipeline are indicated with the prefix “n”; SNPs from the reference guided pipeline are indicated with the prefix “r.” Markers previously associated with QTL resistance in Lebeau et al. (2013) are underlined. The names of 522 markers were removed to ensure good visibility on the map (for more details, see Supplementary Table S3). LG, linkage group; E01–E12, the 12 eggplant chromosomes.

The sequencing of GBS libraries resulted in 662 × 10^6^ paired-end reads of 100 bp (2 × 100). After the cleaning step, a mean of 1.3M reads were demultiplexed for each individual in the RIL population (63% of the reads were discarded). The number of reads in parental lines was 2.5 × 10^6^ for MM738 and 1.9 × 10^6^ for AG91-25. The *de novo* pipeline produced 1,779 filtered SNPs with a mean heterozygosity of 0.04 and a mean missingness rate of 35.6%. The *reference-guided* pipeline produced 890 filtered SNPs with a mean heterozygosity of 0.07 and a mean missingness rate of 27.9%. The two sets of SNPs were merged and redundant SNPs (same physical position) were manually discarded. Good performance of missForest led to a PFC value close to 0. Thus, imputed markers with a PFC > 0.2 were discarded for subsequent analyses. The final data set consisted of 180 genotyped RILs with 1,590 SNPs from the two GBS pipelines and 185 additional molecular markers [amplified fragment length polymorphisms (AFLPs), simple sequence repeats (SSRs), and sequence-related amplified polymorphisms (SRAPs)] previously reported by Lebeau et al. (2013). The genetic map was constructed and based on 867 SNPs, 139 AFLPs, 28 SSRs, and 1 SRAP arranged in 14 linkage groups. Thirty-five percent of the markers were not included in the final map (ungrouped, unrelated or high mean-square contribution markers). Lengths of linkage groups ranged from 37.7 to 156.5 cM and the total length of the map was 1518.1 cM, with an average marker density of 1.47 cM and 22–138 markers per linkage group (**Table 2**). Among the 867 SNP markers (The 90 bp sequences wearing the SNPs), 827 markers gave significant hits on the eggplant reference genome, 199 gave significant hits on the tomato reference genome and 249 gave significant hits on the potato reference genome. Among the 33,873 contigs of the eggplant genome, 595 contigs were anchored in our genetic map, of which 454 were aligned on the tomato genome SL2.40 version. The final dataset, which includes the genetic map and the physical position of SNPs, is provided in Supplementary Table S3. Overall, the data generated a dense new linkage map of eggplant from an intraspecific cross, with 14 linkage groups that have several regions anchored on the tomato genome and produce 12 chromosomes encoded from E01 to E12 (**Figure 1**).

**Table 2:** Statistics of the eggplant [MM738 × AG91-25] RIL population genetic map, including SSR, SRAP, and AFLP from previous work by Lebeau et al. (2013) and newly genotyped SNP markers.

Linkage group (Chromosome)	Length (cM)	Number (No.) markers	No. SNPs	No. SSRs	No. SRAPs	No. AFLPs	Average density^a^ (cM)	Number of gaps^b^ (>10 cM)
1 (E01)	78.7	51	37	2	0	12	1.54	0
13 (E01)	78.7	58	54	0	0	4	1.36	1
2 (E02)	147.7	55	49	2	0	4	2.69	3
3 (E03)	142.1	73	67	3	0	3	1.95	0
4 (E04)	150.6	63	54	4	0	5	2.39	1
5 (E05)	68.9	39	31	2	0	6	1.77	1
14 (E05)	37.7	22	17	0	0	5	1.71	1
6 (E06)	136.7	122	90	5	0	27	1.12	2
7 (E07)	92.5	46	41	1	0	4	2.01	1
8 (E08)	113.5	138	121	1	0	16	0.82	1
9 (E09)	111.6	135	114	3	0	18	0.83	2
10 (E10)	156.5	111	92	2	1	16	1.41	1
11 (E11)	119.6	84	68	3	0	13	1.42	0
12 (E12)	83.1	38	32	0	0	6	2.19	2
Total	1518.1	1035	867	28	1	139	1.47	16

*^a^Number of markers per cM. ^b^Genetic region without marker.*

### Patterns of Bacterial Wilt Resistance in the RIL Population

**FIGURE 2:**
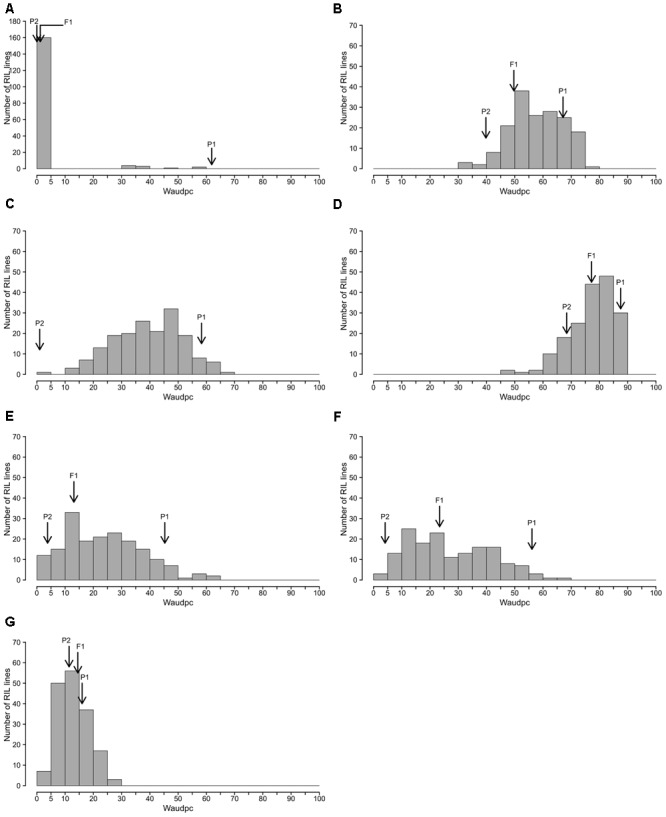
**Frequency distributions of Waudpc in the eggplant [MM738 × AG91-25] RIL population, inoculated with eight *R. solanacearum* species complex strains.** Frequency distribution of Waudpc (area under the disease progress curve, based on the progressive percentage of wilted plants) for strains GMI1000, CMR134, and PSS366 combined in a single analysis **(A)**; combined seasons for strain PSS4 **(B)**; TO10.Indonesia **(C)** T010.Réunion **(D)**; and combined seasons for strains CFBP2957 **(E)**, CFBP3059 **(F)** and CMR34 **(G)**; Arrows indicate the means of susceptible parent P1 (MM738), resistant parent P2 (AG91-25) and their F1 (P1 × P2).

The frequency distributions of Waudpc induced by the eight RSSC strains in the RIL population are shown in **Figure 2**. GMI1000, PSS366 and CMR134 RILs distributions were similar to those reported by Lebeau et al. (2013). Thus, we only present the distribution from the three strains combined (**Figure 2A**). For strain TO10, results are presented separately for Indonesia (**Figure 2C**) and Réunion (**Figure 2D**) because different protocols were used for both assays.

For strains PSS4, CFBP2957, CFBP3059, and CMR34, results for Waudpc variable (**Figures 2B,E–G**) are presented for combined seasons. Each season represents a repetition. For the four strains, frequency distributions for individual seasons are presented in Supplementary Figure S1 (Waudpc). Disease evolution curves of parental lines and the F1 generation are presented in Supplementary Figure S2. Maximal wilting, maximal score, SCOaudpc and CI are not displayed, as these variables are all correlated with Waudpc (0.45 < *R*^2^< 1, depending on strains and seasons). Results indicate four RILs distribution patterns, specific to (i) strains GMI1000, PSS366, and CMR134 (phylotype I); (ii) PSS4 and TO10 (phylotype I); (iii) CFBP2957 and CFBP3059 (phylotypes IIA and III, respectively); and (iv) CMR34 (phylotype IIB).

With the strains GMI1000, PSS366, and CMR134, as published by Lebeau et al. (2013), RILs distribution is discontinuous and skewed toward the highly resistant parent P2 (**Figure 2A**), suggesting that the control of resistance is monogenic.

With strain PSS4, also previously published by Lebeau et al. (2013), the RIL distribution is continuous (**Figure 2B**), P2 is moderately susceptible with a delayed infection and slower pathogenesis than on susceptible P1 (Supplementary Figure S2B). This suggests that a resistance factor influences disease kinetics. When infected with strain TO10, RILs distribution is continuous (**Figure 2C**) and normal (*P* = 1.1 × 10^-01^) for Waudpc in Indonesia. However, it is skewed toward the parent P1 in Réunion (*P* = 1 × 10^-06^) (**Figures 2D**). Parent P1 is highly susceptible in both trials (**Table 3**), particularly in Réunion Island. P2, resistant in Indonesia (**Table 3**), is highly susceptible in the Réunion Island trial, although its disease progression is slower than for P1 (Supplementary Figure S2D), which again suggests that the presence of QTL acts primarily in the early stage of the infection. The broad sense of heritability of the Waudpc is higher in Indonesia (*h*^2^ = 0.71) than in Réunion Island (*h*^2^ = 0.38).

**Table 3:** Mean Waudpc values (with standard errors) for parents P1 (MM738), P2 (AG91-25) and their progenies, obtained with eight *R. solanacearum* species complex strains.

Strain	Location	Parents		Progenies of [P1 × P2]			RILs			
		P1^a^	P2^a^	F_1_	BC_1_P1	BC_1_P2	Mean	V.F^b^	V.F × S^b^	*h*^2c^
GMI1000	Réunion	65.5 (11)	0 (0)	3.5 (0.8)	0.4 (0.4)	0.8 (0.8)	2.9 (0.6)	–	–	–
PSS366	Réunion	53.7 (2.4)	0 (0)	0 (0)	0.4 (0.4)	1.9 (1.9)	2.8 (0.6)	–	–	–
CMR134	Réunion	66.5 (3.2)	0.1 (0.1)	0 (0)	1.4 (0.7)	0 (0)	2.3 (0.5)	–	–	–
Grouping^d^		61.9 (4)	0 (0)	1.2 (0.8)	0.7 (0.3)	0.9 (0.6)	2.7 (0.3)	–	–	–
PSS4	Réunion	67.1 (5.6)	39.8 (10.3)	49.7 (12.5)	59 (8.3)	46.1 (9.2)	57.7 (0.7)	42.36^∗∗∗^	6.12	0.49
TO10	Indonesia	58.3 (3.1)	1 (1)	–	–	–	37.9 (0.8)	131.40^∗∗∗^	–	0.71
	Réunion	87.5 (0.8)	68.4 (4.9)	77.2 (7.8)	84.6 (1.4)	78.7 (5.4)	77.3 (0.5)	25.00^∗∗∗^	–	0.38
CFBP2957	Réunion	45.3 (8.3)	3.8 (1.4)	13.1 (3.9)	31.7 (6.3)	8.8 (3.4)	23.7 (0.7)	133.13^∗∗∗^	12.68	0.73
CFBP3059	Réunion	56.1 (10.1)	4.1 (2)	23.4 (6.6)	35.9 (13.1)	9.9 (5.5)	26.4 (1)	94.94^∗∗∗^	98.56^∗∗∗^	0.48
CMR34	Réunion	16 (1.7)	11.4 (1.3)	14.5 (3.3)	13.5 (2.4)	11.3 (1.1)	12.9 (0.4)	NS	NS	–

*Variance components and heritability are indicated for the eggplant RIL population. Symptoms with CFBP2957, CFBP3059, CMR34, and PSS4 strains were evaluated across two seasons. ^a^P1 and P2 are the MM738 and the AG91-25 parental lines, respectively. ^b^V.F is the estimated variance between RIL families and V.F × S is the estimated variance of RIL families × seasonal interaction. Values are not significant (NS) or significant at ^∗^*P* < 0.05, ^∗∗^*P* < 0.01, ^∗∗∗^*P* < 0.001. ^c^*h*^2^, broad-sense heritability. ^d^Data combined for strains GMI1000, PSS366, and CMR134.*

For both strains CFBP2957 (**Figure 2E**) and CFBP3059 (**Figure 2F**), RILs display a continuous distribution; the parental lines are positioned near the extremes of the frequency distribution. P1 is moderately susceptible, whereas parental line P2 is moderately resistant to both strains (**Table 3**). The values for Waudpc in RIL families did not have a normal distribution (*P*-values of 1.2 × 10^-03^ for CFBP2957; 3.0 × 10^-04^ for CFBP3059). RILs with transgressive phenotypes were observed on both strains (**Figures 2E,F**). The F1 progeny were skewed toward AG91-25, indicating partly dominant inheritance in favor of resistance (Supplementary Figures S2E,F). For both strains, the backcross with parent AG91-25 (BC1P2) was moderately resistant, whereas the backcross with parent MM738 (BC1P1) was moderately susceptible. The ANOVA revealed a highly significant effect of the genotype on Waudpc (*P* < 0.001) (**Table 3**). The genotype × season interaction was not significant in CFBP2957 assay and significant in the CFBP3059 assay (*P* < 0.001; **Table 3**). Waudpc variable is highly heritable with *h*^2^ values of 0.48 and 0.73 for CFBP3059 and CFBP2957 strains, respectively.

Recombinant inbred line distribution is also continuous in the case of strain CMR34. The parental lines are placed in the middle of the distribution (**Figure 2G**). Both are moderately resistant in the first season and partially resistant in the second (Supplementary Figures S1G,H). Waudpc has a normal distribution (*P* = 2.0 × 10^-01^). Values for F1, BC1P2 and BC1P1 progeny (**Table 3**) are all close to those of their parents for both variables. The absence of a genotype effect according to ANOVA indicates that the continuous distributions of the Waudpc variable probably resulted mainly from environmental effects. As the genotype effect was not significant, heritability was not calculated for this strain.

The four patterns of distribution observed for Waudpc indicate the probable co-existence of both complete monogenic and partial polygenic resistance in the RIL population. QTL mapping was undertaken to check this hypothesis.

### A Polygenic Resistance in AG91-25 with a Major QTL and Broad-Range QTLs

**FIGURE 3:**
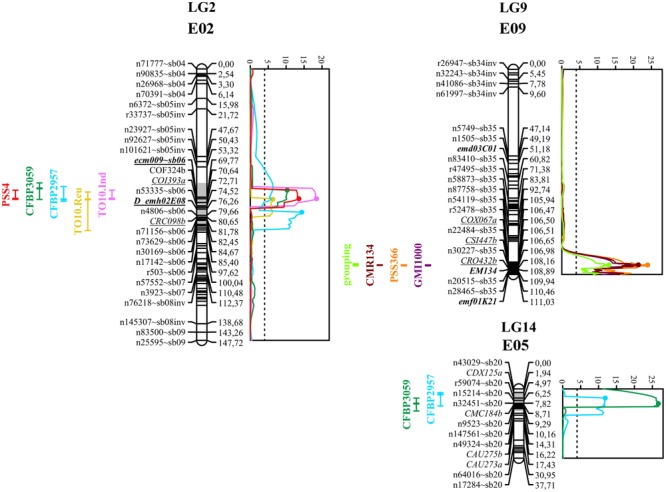
**Graphs of LOD scores, and intervals of *EBWR2, EBWR9, EBWR14* resistance QTLs to *R. solanacearum* species complex positioned on their respective linkage group and chromosome (LG2-E02, LG9-E09, and LG14-E05).** Strains controlled by *EBWR2, EBWR14, EBWR9* QTLs are indicated on the left of each LG. Eggplant/tomato synteny blocks are indicated as “∼sb” at the end of each marker name. Lebeau et al. (2013) markers, linked to resistant QTLs, are underlined. Markers in bold are SSRs. QTL intervals are indicated on the left of each linkage group (LG); LOD curves are plotted on the right of each linkage group (LG). QTL intervals and LOD curves were obtained using CIM for combined seasons (strains CFBP2957, CFBP3059, and PSS4) and individual assays (strains TO10.Indonesia and TO10.Réunion). QTL intervals and LOD curves were obtained with SIM method (strains GMI1000, PSS366, and CMR134) on an individual as well as combined basis. LOD curves were obtained before refining the QTL position with refineqtl R command, whereas QTL intervals were obtained after the refineqtl command.

**FIGURE 4:**
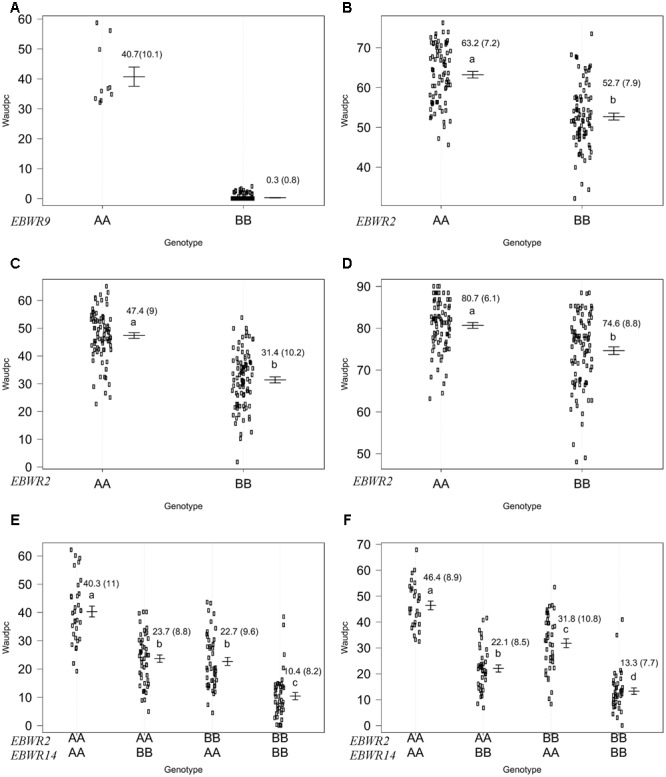
**Phenotypic distribution of genotypes at three eggplant bacterial wilt resistance QTLs for the Waudpc variable.** Allelic effects (allele “A” of susceptible parent P1 and “B” of resistant parent P2) are plotted with the “effectplot” module of R/qtl package at: - *EBWR9* locus, combined data of GMI1000, PSS366, and CMR134 strains **(A)**; - *EBWR2* locus, strain PSS4 **(B)**; strain TO10.Indonesia **(C)**; strain TO10.Réunion **(D)**; - *EBWR2/EBWR14* loci, strain CFBP2957 **(E)**; strain CFBP3059 **(F)**. Means of Waudpc, standard errors (in brackets) and allelic effects ranking (ANOVA followed by LSD test, 95% confidence interval) are indicated. LSD test was not conducted for *EBWR9* locus due to its group’s imbalanced number of RILs. Allelic effects for Waudpc are presented at marker r19023 for *EBWR9*, marker ecm009 for *EBWR2* and at n32451 for *EBWR14*. Heterozygous genotypes are not presented here, as they are very rare in the near fixed RIL population.

Simple Interval Mapping, with both binary (bin) and non-parametric (np) models, was performed on Waudpc values. Strains GMI1000, PSS366, and CMR134 were analyzed separately and in combination (**Table 4**). A single QTL was identified and located on linkage group 9 (Chr. 9) between 106.4 and 106.8 cM (bin model) and between 105.8 and 106.9 cM (np model). LOD scores obtained with the bin model were more homogeneous (13.9–16.5) than those obtained with the np model (13.1–23.9). The QTL, named *EBWR9* according to its position on LG9, co-localized with the COX067a, CRO432b and CSI447b AFLP markers (**Figure 3**) flanking *ERs1* resistance gene, as reported by Lebeau et al. (2013). These results indicate that *EBWR9* and *ERs1* represent the same locus. The genetic region carrying *EBWR9* is extremely distorted in favor of the resistant parent (P2) allele “B.” Among the 170 genotyped and phenotyped RILs, 160 had the “BB” genotype (Waudpc = 0.3 ± 0.8) (**Figure 4A**), whereas 10 had the “AA” genotype (with Waudpc = 40.7 ± 10.1) at the r19023 marker. The locus *EBWR9* was not detected against the other strains of the RSSC.

**Table 4:** QTLs of resistance to three phylotype-I strains of *R. solanacearum* species complex (GMI1000, PSS366, CMR134), detected by Simple Interval Mapping in eggplant [MM738 × AG91-25] RIL population for the Waudpc variable.

Strain	LG^a^	Chr.^b^	QTL^c^	Binary model				Non-parametric model			
				Location^d^ (cM)	Nearest marker	95% CI^e^ (cM)	LOD	Location^d^ (cM)	Nearest marker	95% CI^e^ (cM)	LOD
GMI1000	9	E09	*EBWR9*	106.4	r19023	106.4–106.8	16.5	106.5	COX067a	106.4–106.9	19.5
PSS366	9	E09	*EBWR9*	106.4	r19023	106.4–106.8	16.5	106.5	COX067a	106.4–106.9	23.9
CMR134	9	E09	*EBWR9*	106.5	r52478	106.4–106.7	13.9	106.4	r19023	106.4–106.7	21.4
Grouping	9	E09	*EBWR9*	106.4	r19023	106.4–106.8	16.5	106.5	COX067a	105.8–106.9	13.1

*QTL analysis was carried out for individual strains, as well as for their combined data (Grouping). ^a^Linkage group. ^b^Chromosome corresponding to linkage group. ^c^Name of the QTL: eggplant bacterial wilt resistance (EBWR) followed by linkage group number. ^d^Position of the maximum logarithm of odds score (LOD) in centimorgans (cM). ^e^Bayesian confidence interval.*

Composite Interval Mapping indicated the presence of another resistance QTL on linkage group 14 (E05) (**Table 5**), which was effective against strains CFBP2957 and CFBP3059. For the CFBP2957 strain, a maximum LOD score of 16.3 was observed at 3.0 cM. For the CFBP3059 strain, a maximum LOD score of 27.1 was observed at 8.0 cM. The position of maximum LOD differed slightly according to the strain. However, as the confidence intervals overlapped, the QTL is assumed to be the same and was named *EBWR14*. This QTL was also detected in the analyses season per season, with a maximal LOD position varying from 3 to 12 cM (Supplementary Table S4). This QTL explains 26.1 and 49.6% of the phenotypic variance with the CFBP2957 and CFBP3059 strains, respectively. Thus, it was considered to be a major QTL. The additive effect of the QTL was further analyzed by comparing genotypes homozygous for the P1 (susceptible) allele “A” and homozygous for P2 (resistant) allele “B” at the n32451 marker. The LSD test applied to Waudpc indicated a significant difference (*P*-value < 0.05) between genotypes “AA” and “BB” (Supplementary Table S5). As indicated by the negative additive effect, the resistance-conferring allele of *EBWR14* originated from the resistant parental line P2 (AG91-25).

**Table 5:** QTLs of resistance to four strains of *R. Solanacearum* species complex, detected by Composite Interval Mapping and Haley–Knott regression model in eggplant [MM738 × AG91-25] RIL population for the Waudpc variable.

Strain	Location	LG^a^	Chr.^b^	QTL^c^	Location^d^ (cM)	Nearest marker	95% CI^e^ (cM)	LOD	*R*^2f^	Total R^2g^	Add effect^h^
PSS4	Réunion	2	E02	*EBWR2*	70.6	COF324b	66.0–71.0	13.6	30.8	30.8	–5.2^∗∗∗^
TO10	Indonesia	2	E02	*EBWR2*	70.6	COF324b	66.0–71.0	18.5	38.3	38.3	–7.8^∗∗∗^
	Réunion	2	E02	*EBWR2*	71.0	COF324b	67.0–88.0	6.3	14.9	14.9	–3.3^∗∗∗^
CFBP2957	Réunion	2	E02	*EBWR2*	71.0	COF324b	64.0–72.0	16.7	27.0	49.4	–7.3^∗∗∗^
		14	E05	*EBWR14*	3.0	CDX125a	2.0–9.3	16.3	26.1		–7.3^∗∗∗^
CFBP3059	Réunion	2	E02	*EBWR2*	65.0	ecm009	62.0–71.0	9.3	12.8	58.8	–6.2^∗∗∗^
		14	E05	*EBWR14*	8.0	n32451	5.0–12.0	27.1	49.6		–10.4^∗∗∗^

*QTL analysis was carried out on two seasons combined data for PSS4, CFBP2957, CFBP3059 and CMR34 strains, and for individual location for TO10 strain. ^a^Linkage group. ^b^Chromosome corresponding to linkage group. ^c^Name of the QTL: eggplant bacterial wilt resistance (EBWR) followed by the linkage group number. ^d^Position of the maximum logarithm of odds score (LOD) in centimorgans (cM). ^e^Bayesian confidence interval. ^f^*R*^2^: Estimates of the proportion of phenotypic variance (percentage) explained by the QTL detected. ^g^Estimate of the total proportion of phenotypic variance explained by the additive model. ^h^Additive effect: Positive values mean that the resistance allele comes from the P1 (MM738) parent, while negative values mean that the resistance allele comes from the P2 (AG91-25) parent.*

Another QTL (**Table 5**) was detected by CIM on linkage group 2 (E02) for strains CFBP3059, CFBP2957, TO10, and PSS4 (Waudpc). Maximum LOD was positioned between 65 and 71 cM, and the LOD scores varied from 6.3 to 18.5 (**Table 5**). This QTL explains Waudpc phenotypic variance for TO10.Indonesia (38.3%), TO10.Réunion (14.9%), CFBP2957 (27%), CFBP3059 (12.8%), and PSS4 (30.8%). For each strain, the additive effect is always negative: this indicates that the resistance comes from the P2 resistant parent. A significant difference between genotypes “AA” and “BB” for Waudpc was found at this QTL (*P*-value < 0.05, Supplementary Table S5) for strains PSS4 (**Figure 4B**), TO10.Indonesia (**Figure 4C**), and TO10.Réunion (**Figure 4D**). As the LOD curve on linkage group 2 displays two peaks between 50 and 80 cM (**Figure 3**), the presence of a second QTL was checked using the stepwise procedure implemented in R/qtl. However, no other QTL was found on LG2 (data not shown). Thus, we concluded that only one QTL, called *EBWR2*, was present. It co-localizes with ecm009 and COI393a markers, which are reported by Lebeau et al. (2013) to be linked to the QTL effective against PSS4 strain. Our results confirm the presence of this QTL and demonstrate that it is broad-spectrum because it is also effective against three other strains (TO10, CFBP2957, and CFBP3059).

Strains CFBP3059 and CFBP2957 are controlled by both *EBWR2* and *EBWR14* loci (**Table 5**). No significant epistatic digenic interaction between *EBWR2* and *EBWR14* was found (fitqtl function of R/qtl). For both strains, the RIL group that is homozygous for P2 resistant alleles at both *EBWR2*/*EBWR14* loci (i.e., allele “B” at *EBWR2* and “B” at *EBWR14*, i.e., genotypes BB/BB) display significantly lower Waudpc means (**Figures 4E,F** and Supplementary Table S5) than the group with only one resistant allele at any locus (genotypes BB/AA or AA/BB). The latter genotypes do not differ from each other for the CFBP2957 strain (**Figure 4E**). In contrast, for strain CFBP3059 (**Figure 4F** and Supplementary Table S5), genotypes AA/BB display a significantly lower Waudpc means than genotypes BB/AA.

Lastly, the present study did not detect a QTL controlling the strain CMR34 in any of the analyses carried out (CIM or stepwise method; and regardless of whether the seasons were considered separately or together).

In short, QTLs analyses identify three QTLs in the RIL population. *EBWR9* has a major effect but is only detected with three strains (GMI1000, PSS366, and CMR134) belonging to the phylotype I. *EBWR2* and *EBWR14* are both involved in the resistance to strains CFBP2957 and CFBP3059, but they do not interact. *EBWR2* is the sole QTL detected with PSS4 and TO10 strains. Lastly, no QTL controls CMR34.

### Anchoring Resistance QTLs on the Tomato Physical Map

The three different QTLs of resistance to RSSC were mapped on the eggplant genome thanks to the anchor SNPs from our new linkage map. The corresponding eggplant/tomato synteny blocks were assigned according to the position of eggplant contigs (Hirakawa et al., 2014). The corresponding tomato physical positions are from the latest SL2.50 tomato genome version. The physical position on the older SL2.40 tomato genome version can be found in Supplementary Table S3, as all eggplant contigs were anchored on this genome version by Hirakawa et al. (2014). *EBWR9*, the major QTL controlling strains GMI1000, PSS366, and CMR134, was located on a section of eggplant and tomato Chr. 9, matching eggplant/tomato synteny block 35. According to the largest confidence interval obtained from QTL analysis (105.8 to 106.9 cM), *EBWR9* is located approximately between 69.4 and 70.5 Mbp on tomato Chr. 9.

*EBWR14*, the major QTL that controls the strains CFBP2957 and CFBP3059, was located on a section matching eggplant Chr. 5 and tomato Chr. 12. This section is part of the synteny block 20 (**Figure 3** and Supplementary Table S3). The *EBWR14* confidence interval varies from 2 to 12 cM, depending on the strains (CFBP2957 and CFBP3059). This second section matches the 64.4–66.9 Mbp region of the tomato Chr. 12.

Broad spectrum QTL *EBWR2*, which controls strain PSS4, TO10, CFBP2957, and CFBP3059, was located on the eggplant and tomato Chr. 2, corresponding to the eggplant/tomato synteny blocks sb05inv and sb06 (**Figure 3** and Supplementary Table S3). The *EBWR2* confidence interval varies from 61 to 79 cM and corresponds to a physical interval of 38.3 to 45.9 Mbp of the tomato Chr. 2. However, the presence of two large gaps and the lack of anchor markers in the QTL interval complicate the definition of its physical interval.

Thanks to the dense and anchored eggplant genetic map that we set up, the three QTLs of resistance to RSSC were located at physical sections on the eggplant/tomato synteny blocks. *EBWR2* and *EBWR14* are still positioned with a large interval; the densification of good quality markers associated to these QTLs is necessary before conducting a candidate gene approach. On the contrary, the *EBWR9* locus is saturated with markers and adding new markers will not help narrow the interval. Therefore, we immediately decided to look for candidate genes within the *EBWR9* locus.

### Physical Interval Containing *EBWR9* QTL and Search for Candidate Genes

Markers positioned at the *EBWR9* locus were reorganized according to their physical position on the eggplant/tomato synteny block 35. Among the 170 phenotyped and genotyped RILs, 151 had a fixed haplotype with the P2 (AG91-25) resistant allele (B), seven had a fixed haplotype with the P1 (MM738) susceptible allele (A), eight had heterogeneous regions (heterogeneous inbred families, HIFs) and only four families had fixed recombinant haplotypes (**Table 6**). Resistant families (phenotype R) should be homozygous for the “B” allele (light gray box), whereas susceptible families (phenotype S) should be homozygous for the “A” allele (black boxes) at the *EBWR9* locus. Accordingly, *EBWR9* was positioned between the Sme2.5_00457.1 and Sme2.5_00934.1 contigs of eggplant. This region spans a physical distance of 1.8 Mbp between 69.40 and 71.17 Mbp of tomato Chr. 9, corresponding to 3.0 Mbp of the potato Chr. 9. Among the eight HIFs, four have a resistant phenotype and are homozygous for the P2 “B” allele within the refined locus limits (**Table 6**). The four other RIL families segregated for these markers included two that were resistant and two that were susceptible.

**Table 6:** Fine physical position of *EBWR9*, an eggplant major resistance QTL to 3 phylotype-I strains.

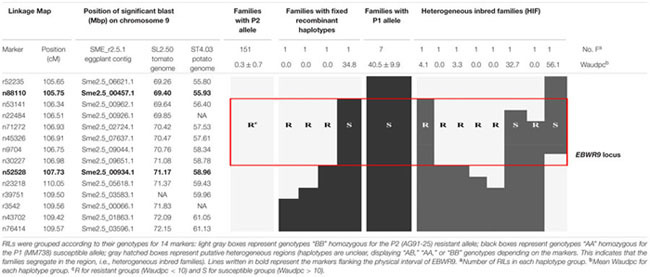

Seven R-gene analog (RGA) fragments of the P1 (MM738) transcriptome were found in the LG9-*EBWR9* refined region. The list of the transcripts and their corresponding tomato orthologous genes are described in **Table 7**. Only two genes of the NBS-LRR class were found in the region, the singlet__11792 on Sme2.5_04376.1 and the singlet__6456 on Sme2.5_00239.1. The singlet__40855, matching the tomato Solyc09g091260.2 RLK gene, was found on eggplant contig Sme2.5_00507.1. The singlet__57090 and singlet__10906, mapped on the same eggplant contig Sme2.5_00341.1 probably belong to the same gene and correspond to two different exons of the RLK tomato Solyc05g009040.2 gene. Both Singlet__34133 and singlet__13069, mapped on the Sme2.5_00934.1 contig, probably belong to another RLK gene, very similar to the tomato Solyc09g091990.2 gene. All seven transcripts listed in the table had different SNP alleles and predicted genes products between the MM738 and AG91-25 parental lines (Supplementary Table S6). Moreover, all the eggplant contigs listed in **Table 7** contain markers that have been mapped on LG9: Sme2.5_04376.1 contains n93417, which is mapped at 106.33 cM; Sme2.5_00239.1 contains r8676, n14281, n9304, n62318, and r8690, which are mapped, respectively, at 106.22, 106.26, 106.61, 106.69, and 106.79 cM; Sme2.5_00507.1 contains n59329, mapped at 105.83 cM; Sme2.5_00341.1 contains n53260 mapped at 107.22 cM, Sme2.5_00934.1 contains n52528 and n136813 mapped, respectively, at 107.73 and 107.04 cM (Supplementary Table S3). Thus, we can assume that the five candidate genes described in **Table 7** are all co-segregating with the major QTL *EBWR9*. Only one RGA gene (Solyc09g091400.2), found in the tomato transcriptome, which matches the locus EBWR9 in eggplant, is absent (not expressed) in the P1 parent (MM738) transcriptome. This gene is a member of the RLK family and could be considered as a candidate gene for BW-resistance in eggplant. Finally, RGA genes were not specifically present in *EBWR9* locus of the resistant parent P2.

**Table 7:** List of putative candidate genes for resistance to bacterial wilt retrieved from the eggplant P1 (MM738) parental line transcriptome in the *EBWR9* physical region and their positions on the eggplant genome.

Position of MM738 transcripts on the SME_r2.5.1 eggplant genome				Significant hit on the tomato genome			
Transcript ID^a^	Eggplant genome contig ID	Position start	Position end	Tomato gene ID	Identity (%)	Blast e-value	Gene class
singlet__11792	Sme2.5_04376.1	2044	2462	Solyc09g064680.1	83.82	4.0 × 10^-27^	NBS-LRR
singlet__6456	Sme2.5_00239.1	117683	116834	Solyc09g090620.1	86.83	1.0 × 10^-146^	CC-NBS-LRR
singlet__40855	Sme2.5_00507.1	98065	103689	Solyc09g091260.2	93.64	0.0	RLK
singlet__57090	Sme2.5_00341.1	1051	1487	Solyc05g009040.2	90.11	1.0 × 10^-147^	RLK
singlet__10906	Sme2.5_00341.1	1632	2123	Solyc05g009040.2	92.67	0.0	RLK
singlet__34133	Sme2.5_00934.1	41969	42396	Solyc09g091990.2	95.48	1.0 × 10^-163^	RLK
singlet__13069	Sme2.5_00934.1	43828	42504	Solyc09g091990.2	91.40	0.0	RLK

*The corresponding orthologs genes found in the tomato transcriptome ITAG2.4 are indicated. ^a^Transcripts from P1 (MM738) susceptible parent.*

*Ralstonia solanacearum* species complex resistance genes *RRS1, RPS4, ERECTA, RD19* and *wat1* in *A. thaliana* and *RE-bw* in *S. melongena* were mapped on tomato and eggplant genomes with the Blastn tool. No significant hits were found for the *RRS1* and *RPS4* genes. *ERECTA* had a significant hit on Sme2.5_05203.1 of the eggplant Chr. 8 and on the tomato Chr. 8 at 49 Mbp. *RD19* had a significant hit on the Sme2.5_00650.1 of the eggplant Chr. 1 and on the tomato Chr. 1 at 96.8 Mbp. Two significant hits were found for *RE-bw* on tomato chromosomes: one on Chr. 11 at 55.3 Mbp and the other on Chr. 8 at 2.2 Mbp. Best significant hits (*E*-value > 1 × 10^-30^) for *RE-bw* were found on the eggplant contigs Sme2.5_05713, Sme2.5_01979.1, Sme2.5_00683.1, Sme2.5_16002.1 (chromosome E12/syntenic block sb54inv), Sme2.5_15564.1 (E11/sb46inv), Sme2.5_08973.1, Sme2.5_13120.1 (E12/sb55inv) and Sme2.5_08697.1 (E01/sb01). The *wat1* gene gave one significant hit on the Sme_00003.1 of eggplant Chr. 4 and one significant hit on the tomato Chr. 4 at 65 Mbp.

Based on these results, none of the RRSC resistance genes cloned so far significantly hit the *EBWR9* E09/sb35 region.

## Discussion

### A New Dense Anchored Map of the [MM738 × AG91-25] RIL Population

The map of the intraspecific RIL population previously published consisted of 119 markers spread over 18 linkage groups. The markers were mainly AFLP and the map could not be traced back to the 12 chromosomes of tomato or eggplant. Thanks to the use of GBS, this map has been significantly improved. With 867 new SNPs, it is now anchored on the physical chromosomes of tomato and potato. The large number of sequenced loci resolved the initial problem of low polymorphism. The anchor SNPs are positioned on 50 of the 56 reference eggplant/tomato synteny blocks. Thus, we assume that this map provides a comprehensive overview of the eggplant genome. GBS usually produces a great deal of missing data, particularly when using an effective cutting enzyme, such as ApeKI. However, the genetic map is almost consistent with the physical order of markers on eggplant and tomato chromosomes, apart from a few inconsistencies for the position or order of some markers. The map still has some large gaps, particularly two that are close to *EBWR2*. In future, markers should be added to these regions to ensure greater density coverage and finer mapping of this broad-spectrum QTL.

Distorted markers occurred in some chromosomic regions, particularly on Chr. 8 and 9. Segregation distortions are frequently observed in mapping populations and the amount of distorted markers has been correlated to the taxonomic divergence between parental lines (Zhang et al., 2003). P2 (AG91-25), the resistant parent of the RIL population, is a line that comes from a complex breeding program involving one accession of *S. aethiopicum* Aculeatum Group (resistant to BW) and several *S. melongena* accessions (Ano et al., 1991). We assume that *EBWR9*, the major resistance QTL on Chr. 9, is probably positioned on an *S. aethiopicum* introgressed segment. This introgression could explain the higher polymorphism and the segregation distortion rate in this region. In future, the species origin of the chromosomal segment harboring *EBWR9* could be confirmed by comparing the transcriptome of P2 (AG91-25) to the reference transcriptomes of *S. melongena* (Hirakawa et al., 2014) and *S. aethiopicum* (Gramazio et al., 2016).

### RSSC Resistance of AG91-25 Is a Complex System with Both a Major Specific QTL and Broad-Spectrum QTLs

Three QTLs controlling resistance to RSSC were identified in the [MM738 × AG91-25] RIL population. The first QTL, *EBWR9*, located on Chr. 9, matches the major gene *ERs1* (Lebeau et al., 2013). *EBWR9* is detected with GMI1000, PSS366, and CMR134 (phylotype I strains). The Waudpc variable controlled by this QTL has a qualitative distribution, which suggests a monogenic control of the resistance. This QTL has a very strong effect: all RIL lines with the resistant parent allele (B) are highly resistant. The QTL was not detected with the virulent PSS4 and TO10 (also phylotype I strains), nor with strains belonging to other phylotypes (CFBP2957, CFBP3059, and CMR34). This result suggests a possible phylotype-specificity of *EBWR9*, together with a strain-specificity within phylotype I. A relationship between BW-resistance QTLs and phylotypes has already been proposed in tomato (Carmeille et al., 2006). Two major QTLs, *bwr-6* and *bwr-12*, were identified in this study. The moderate effect of *bwr-6* on a large spectrum of strains suggested that several phylotypes were controlled. The major effect of *bwr-12* seemed to be phylotype I-specific. Therefore, we suggest that there are probable similarities between eggplant and tomato BW-resistance mechanisms.

The second major QTL that we identified in the RIL population, *EBWR14*, is positioned on eggplant Chr. 5. Interestingly, *EBWR14* is located in a region of tomato Chr. 12 on the same chromosome as *bwr-12.* However, *bwr-12* was located between the SLM12-10 and SML12-2 markers (Carmeille et al., 2006; Wang et al., 2013), within a physical region between 2.78 and 3.30 Mbp. This region belongs to the eggplant/tomato synteny block 51. As *EBWR14* is located on synteny block 20, both QTLs are distinct, although they are located on tomato Chr. 12.

The third QTL, *EBWR2*, positioned on Chr. 2, partially controls strains belonging to phylotypes I (strains PSS4, T010), phylotype IIA (CFBP2957) and phylotype III (CFBP3059). It is not detected with three other phylotype I strains (GMI1000, PSS366, and CMR134). Its effect is minor or intermediate, depending on strains and environmental conditions. Its confidence interval is large (61–79 cM) and varies depending on the strains. The two peaks in the LOD curves indicate that the presence of two closed QTLs is highly likely. Interestingly, Lebeau et al. (2013) detected one or two QTLs on eggplant LG13 (matching Chr. 2 in our study), depending on the analysis method used (SIM and CIM, respectively). In tomato, a QTL with a large confidence interval was subdivided into two QTLs, each acting at different stages of the infection (Mangin et al., 1999). Coupled QTLs such as this, for which the maximum LOD score is located between the two QTLs, have been reported as “ghost QTLs” (Martinez and Curnow, 1992; Broman and Speed, 1999). We used the stepwise automated procedure of R/qtl (multiple QTL mapping method) to prevent the “ghost” QTL detection. However, we did not detect the presence of a second QTL on Chr. 2. The existence of a second QTL could be checked in future, after the region has been densified with high quality markers (Nilsson et al., 1999; Li et al., 2010; Stange et al., 2013), developed from Kompetitive Allele Specific PCR (KASP; Semagn et al., 2014) or high resolution melting PCR (HRM; Gundry et al., 2003). The size of the mapping population could also be a major limitation to resolve linked QTLs and should be increased in future research.

### *EBWR9*, Close to a Hot Spot of Resistance Genes, Is Distinct from RSSC Resistance Cloned Genes

**FIGURE 5:**
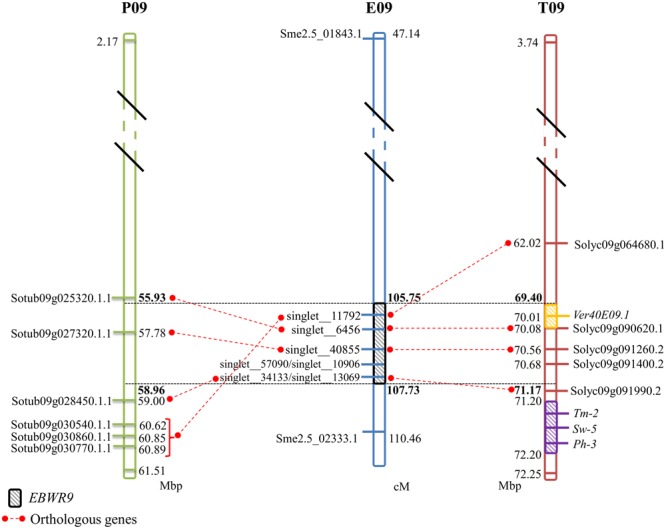
**Comparison between eggplant linkage group E09, tomato chromosome 9 (T09), and potato chromosome 9 (P09) for the synteny block 35, where *EBWR9* major QTL for bacterial wilt resistance is located.** Position of *EBWR9* and the candidate genes identified in the P1 (MM738) transcriptome are indicated on E09. Physical position of candidate genes from the ITAG2.4 transcriptome and QTLs/genes controlling tomato resistance to *Verticillium dahliae resistance* (*Ver40E09.1*), tomato spotted wilt virus (*Sw-5*), tomato mosaic virus (*Tm-2*) and *Phytophthora infestans* (*Ph-3*) identified in previous studies are indicated on T09. Orthology between eggplant, tomato and potato genes (ST1.0 transcriptome) is indicated by red dotted lines. Map distances (cM) are shown on the right of E09 and physical distances (Mbp) are shown on the right of P09 and on the left of T09. Position of the *EBWR9* interval on P09, T09, and T09 are printed in bold font. Distances are not proportional.

No significant hits were found between *EBWR9* and known BW-resistance genes. However, at this extremity of the long arm of Chr.9, three other resistance loci have been reported in tomato. They confer resistance against tomato spotted wilt virus (*Sw-5*), tomato mosaic virus (*Tm-2*) and *Phytophthora infestans* (*Ph-3*). *Sw-5* and *Tm-2* have been located between 71.2 and 72.2 Mb and *Ph-3* between 71.4 and 71.5 Mb on the tomato Chr. 9 (Zhang et al., 2013, 2014; Andolfo et al., 2014; Panthee et al., 2015). We located *EBWR9* between 69.40 and 71.17 Mbp, just before the cluster of *Sw-5, Tm-2*, and *Ph-3* resistance genes (**Figure 5**). Furthermore, *EBWR9* was mapped at the same physical position as the minor QTL for *Verticillium dahliae* resistance (*Ver20E09.1*, located at 70.14 Mbp on tomato; Toppino et al., 2016). The population used for mapping *V. dahliae* resistance derives from a cross between the *S. melongena* susceptible 67/3 line and the 305E40 resistant line, which has an *S. aethiopicum* ancestor in its pedigree similar to our P2 *(*AG91-25) (Ano et al., 1991). These results suggest that the *EBWR9* and *Ver20E09.1 V. dahlia* resistance QTLs could be the same and may both originate from an *S. aethiopicum* introgression fragment.

Overall, these results suggest that the ends of tomato and eggplant Chr. 9 are enriched in disease resistant genes. This region is, therefore, of special interest for breeding cultivars resistant to diverse pathogens.

### A First Step toward Cloning EBWR9

The region harboring *EBWR9* major QTL is distorted in favor of P2’s resistant allele “B” (AG91-25). In this region, genetic and physical orders display some discrepancies. To reduce the QTL interval, we rearranged the markers according to their physical position and refined the confidence interval by comparing phenotypes from the haplotype groups. The physical position of *EBWR9* was defined within a 1.77 Mbp interval on tomato Chr. 9. In this interval, we did not found RGA which are uniquely expressed in the resistant line P2. However, the published transcriptome of the susceptible line P1 (Sarah et al., 2016) enables the detection of seven transcripts encoded by this region, five of which correspond to RGA, which significantly hit the tomato transcriptome. All five genes are expressed in both P1 (MM738) and P2 (AG91-25), but their alleles differ. Four of them are located on the eggplant/tomato synteny block 35 and two below the *EBWR9* region (**Figure 5**). Each transcript contains non-synonymous substitutions which induce variation in the predicted resistance protein. Therefore, they are candidate genes for BW-resistance in eggplant. In future, it should be possible to determine the differential expression of the five genes during GMI1000 strain infection, by using quantitative reverse transcription-PCR to test whether or not they are involved in defense mechanisms. However, a candidate gene found in the tomato transcriptome is absent from the eggplant transcriptome. The expression of resistance genes is not always constitutive. Resistance genes can be induced or drastically up-regulated after the plant has recognized the pathogen (Yoshimura et al., 1998; Levy et al., 2004). Moreover, 13% of the 237 tomato genes found inside the *EBWR9* locus are not annotated. Thus, our list of candidate genes is not exhaustive and will probably be enlarged in future.

Heterogeneous inbred families, i.e., RILs with residual heterozygosity at the *EBWR9* locus, will be used for fine mapping the putative resistance gene(s) via the creation of near isogenic lines (NILs) (Tuinstra et al., 1997; Yeri et al., 2014). Four HIFs from our RIL population are particularly interesting because their heterogeneity within the refined *EBWR9* locus will make it possible to reduce the physical interval of *EBWR9.* Even if only five candidate genes have been found in the *EBWR9* region, their number must be reduced in order to characterize the gene underlying the *EBWR9* QTL. A number of the 41 SNPs, identified within the *EBWR9* physical interval, deserve to be validated by using the KASP or HRM SNP genotyping system so that they can subsequently be used on the NILs for fine mapping. New SNPs residing in the five RGA could also be designed from their transcriptomic sequences and fine mapped in the HIFs.

### Breeding for Broad Spectrum and Durable Resistance to RSSC

Strains of RSSC have a worldwide distribution and exhibit a very large host range. Eggplant has the disadvantage of being a potential host for strains belonging to the four phylotypes of RSSC. Thus, in order to breed eggplant for resistance to BW, we need to further our understanding of the molecular and genetic bases of resistance controlling the diversity of RSSC strains. We dissected the BW-resistance of the P2 (AG91-25) line, by mapping its resistance factors in a RIL population. Three resistance QTLs control strains belonging to one or more phylotypes. *EBWR9* provides the highest level of resistance, although its range of efficiency is limited to phylotype I strains (GMI1000, PSS366, and CMR134). *EBWR2* has the largest range of efficiency because it is effective against strains of phylotypes I (PSS4 and TO10), IIA (CFBP2957) and III (CFBP3059). *EBWR14* only controls strains of phylotype IIA and III. Therefore, both *EBWR2* and *EBWR14* are useful for breeding resistant varieties in areas where phylotypes IIA and III are present. Our phenotyping tests were carried out under greenhouse conditions with very high inoculation pressure. Therefore, we may have under-estimated the efficiency of both QTLs in real cropping conditions. Furthermore, assays conducted with TO10 strain in two different places (greenhouses in Indonesia and Réunion Island) revealed a strong interaction between phenotype (P) × environment (E). The P2 (AG91-25) parent showed different phenotypes, while the P1 (MMM738) parent remained susceptible in both environments. The environmental influence on P2 resistance mechanisms was also detected in QTL analysis. In Indonesia, *EBWR2* was detected from day 14 after the inoculation (dai) until the end of the assay. However, in Réunion, *EBWR2* was only detected between 7 and 14 dai (data not shown). These results cannot be correlated to the inoculum pressure given that the quantity of bacterium delivered in each plant was superior in Indonesia than in Réunion. The results underline the importance of conducting breeding programs in real environmental conditions. By combining *EBWR2, EBWR14*, and *EBWR9*, breeders could obtain cultivars with a large spectrum of BW-resistance. Despite the medium-term risk of the emergence of strains that bypass the major resistance controlled by *EBWR9*, this QTL is of immediate value for breeders because it can easily be introgressed in commercial cultivars using Marker Assisted Selection. However, we advise combining *EBWR9* with other major genes or QTLs for resistance in order to avoid rapid resistance breakdown (Brun et al., 2010; Djian-Caporalino et al., 2014; Barbary et al., 2016). Furthermore, *EBWR9* has already been demonstrated to be ineffective against at least two strains of phylotype I. Therefore, it is important to identify other sources of resistance in eggplant’s natural genetic diversity, with a broader scope in terms of controlling bacteria genetic diversity, which can be used through a pyramiding strategy of resistance genes. To reach this objective, conducting a genome-wide association study on a core collection of eggplant accessions may be an effective way to identify additional loci involved in other sources of resistance.

The high level of resistance conferred by *EBWR9*, together with its strain specificity, suggests the existence of a gene-for-gene relationship of an R/Avr type. Cloning *EBWR9* requires further time-consuming and expensive experiments. The identification of the corresponding avirulence effector is promising, since effectors can be used to determine functional redundancy in plant germplasm. This could help breeders choose complementary sources of resistance (Vleeshouwers and Oliver, 2014). Furthermore, the effectors matching *EBWR9* could be used to find its homologs in Solanaceae in both cultivated and wild germplasm, particularly potato, tomato, and capsicum pepper. In this respect, the ripP2 and ripAX2 are type-III effectors, which are of great interest because of their association with avirulence on AG91-25 (Pensec et al., 2015).

## Conclusion

A complex polygenic system of resistance based on three BW-resistance QTLs was dissected in the [MM738 × AG91-25] RIL population. Thanks to the new densified and anchored linkage map, the physical positions of the QTLs were estimated. The QTL that provides the highest level of resistance is *EBWR9*, which is highly effective against GMI1000, PSS366, and CMR134 phylotype I strains. However, it is ineffective against virulent strains of phylotype I and strains of phylotypes II and III. Two other QTLs were detected, namely *EBWR2* and *EBWR14*. They drastically reduce wilting symptoms and stem colonization by strains that are not controlled by *EBWR9*, when both resistant alleles are combined. Thus, AG91-25 is an outstanding source of resistance to BW, which should lead to the development of broad-spectrum resistant cultivars. The GBS sequences flanking the QTLs can be used to develop breeder-friendly markers (e.g., using the KASP or HRM method), which are indispensable for a rapid transfer of BW-resistance in commercial cultivars. In addition to their direct applicability to breeding, our results provided the first step toward the cloning of *EBWR9* (previously known as *ERs1*). *EBWR9* is located at the end of Chr. 9, a region reported to be rich in disease-resistance factors. A cluster of R-genes can be found close to its physical interval, but none of them co-localize with *EBWR9*. However, seven transcripts from the susceptible MM738 line, corresponding to five annotated R-genes, were found in this region. These transcripts will be characterized in the near future. Ultimately, our results should (i) provide a better understanding of the interaction between RSSC genetic diversity and eggplant resistance QTLs, (ii) lead to the investigation of the possible R/Avr system involved in AG91-25 resistance, and (iii) contribute to the development of breeding strategies that encourage a sustainable approach to controlling this devastating pathogen.

## Author Contributions

M-CD, BR, EW, and JD designed the study. M-CD produced the mapping population seeds. SS carried out the phenotyping experiments. CS performed transcriptomic experiments. SS, CJ, and CS performed bioinformatics analyses. SS put together the linkage map and conducted the QTL analysis. SS drafted the manuscript. CJ, CS, JD, EW, and M-CD contributed to writing and finalizing the paper. All authors have read and approved the final manuscript.

## Conflict of Interest Statement

The authors declare that the research was conducted in the absence of any commercial or financial relationships that could be construed as a potential conflict of interest.

## Footnotes

1 https://sourceforge.net/projects/gbsbarcode/

2 ftp://ftp.sgn.cornell.edu/tomato_genome/annotation/ITAG2.4_release

3 http://arcad-bioinformatics.southgreen.fr/node/17

4 www.solgenomics.net
