# Supplementary material for: Eggplant Resistance to the Ralstonia solanacearum Species Complex Involves Both Broad-Spectrum and Strain-Specific Quantitative Trait Loci
Source: Front Plant Sci. 2017 May 19;8:828. doi: 10.3389/fpls.2017.00828 (PMC5437220; doi:10.3389/fpls.2017.00828)
Supplement: Supplementary file 2 [file Presentation_1.PDF]

Supplementary Material

Eggplant resistance to the *Ralstonia solanacearum* species complex involves both broad-spectrum and strain-specific quantitative trait loci

Sylvia Salgon[1,2,3*], Cyril Jourda[1], Christopher Sauvage[4], Marie-Christine Daunay[4], Bernard Reynaud[1,3], Emmanuel Wicker[1,5], Jacques Dintinger[1]

*** Correspondence:** Sylvia Salgon: sylvia.salgon@gmail.com; Jacques Dintinger: jacques.dintinger@cirad.fr.

1 Supplementary Tables

Table S1. List of barcodes associated to each eggplant sample used for Genotyping by sequencing.

Samples	Plate	Barcode	Sample Type	Comments
blank	1	TTCCTGAA	Negative control	Empty[a]
blank	2	TTCCTGAA	Negative control	Empty
G003	1	GCTTA	RIL	-
G007	1	AACGCCT	RIL	-
G009	1	AGGC	RIL	-
G011	1	TCGTT	RIL	Discarded[b]
G014	1	TGGCTA	RIL	-
G017	1	TGCTGGA	RIL	-
G019	1	TGCA	RIL	-
G020	1	AGCCC	RIL	-
G021	1	CTTCCA	RIL	-
G023	1	AATATGC	RIL	-
G025	1	GATC	RIL	-
G026	1	ACCTAA	RIL	-
G028	1	ACGTGTT	RIL	-
G029	1	AACCGAGA	RIL	-
G030	1	ACTA	RIL	-
G033	1	GTATT	RIL	-
G034	1	GAGATA	RIL	-
G036	1	ACGACTAC	RIL	-
G038	1	TCAC	RIL	-
G041	1	ATATGT	RIL	-
G042	1	ATTAATT	RIL	-
G043	1	ACAGGGAA	RIL	-
G044	1	CAGA	RIL	-
G047	1	CTGTA	RIL	-
G048	1	ATGCCT	RIL	-

Table S1. Continued

Samples	Plate	Barcode	Sample Type	Comments
G052	1	GGTGT	RIL	-
G056	1	AGGAT	RIL	-
G058	1	ATCGTA	RIL	-
G059	1	ATTGGAT	RIL	-
G063	1	ACGTGGTA	RIL	-
G065	1	AACT	RIL	-
G066	1	ACCGT	RIL	-
G067	1	TATTTTT	RIL	-
G068	1	TAGCATGC	RIL	-
G071	1	ATTGA	RIL	-
G072	1	CATCGT	RIL	-
G074	1	CATAAGT	RIL	-
G075	1	CCATGGGT	RIL	-
G079	1	GCGT	RIL	-
G083	1	GTAA	RIL	-
G084	1	CTTGCTT	RIL	-
G085	1	AGTGGA	RIL	-
G086	1	CATCT	RIL	-
G087	1	CGCGGT	RIL	-
G089	1	CGCTGAT	RIL	-
G090	1	CGCGGAGA	RIL	-
G091	1	TGCGA	RIL	-
G092	1	GGTTGT	RIL	-
G093	1	ATGAAAC	RIL	-
G094	1	TAGGCCAT	RIL	-
G095	1	CCTAC	RIL	-
G096	1	CTATTA	RIL	-
G097	1	CGGTAGA	RIL	-
G098	1	CGTGTGGT	RIL	-
G099	1	CGAT	RIL	-
G100	1	CCAGCT	RIL	-
G104	1	AAAAGTT	RIL	-
G106	1	TGCAAGGA	RIL	-
G107	1	GAGGA	RIL	-
G108	1	GCCAGT	RIL	-
G109	1	CTACGGA	RIL	-
G110	1	GCTGTGGA	RIL	-
G111	1	CGCTT	RIL	-
G113	1	TTCAGA	RIL	-
G114	1	GAATTCA	RIL	-
G117	1	TGGTACGT	RIL	-
G118	1	GGAAC	RIL	-

Table S1. Continued

Samples	Plate	Barcode	Sample Type	Comments
G119	1	GGAAGA	RIL	-
G121	1	GCGGAAT	RIL	-
G122	1	GGATTGGT	RIL	-
G123	1	TCACC	RIL	-
G124	1	TAGGAA	RIL	-
G127	1	GAACTTC	RIL	-
G128	1	TCTCAGTC	RIL	-
G129	1	GTCAA	RIL	-
G130	1	GTACTT	RIL	-
G131	1	TAGCGGA	RIL	-
G132	1	GTGAGGGT	RIL	-
G135	1	CTAGC	RIL	-
G136	1	GCTCTA	RIL	-
G137	1	GGACCTA	RIL	-
G138	1	CCGGATAT	RIL	-
G139	1	TAATA	RIL	-
G140	1	GTTGAA	RIL	-
G142	1	TCGAAGA	RIL	-
G144	1	TATCGGGA	RIL	-
G145	1	ACAAA	RIL	Discarded
G146	1	CCACAA	RIL	-
G147	1	GTCGATT	RIL	Discarded
G148	1	CGCCTTAT	RIL	-
G150	1	TACAT	RIL	Discarded
G151	1	TAACGA	RIL	-
G152	1	TCTGTGA	RIL	-
G153	2	CTCC	RIL	-
G154	2	TTCTC	RIL	-
G156	2	GCTTA	RIL	-
G157	2	AACGCCT	RIL	-
G158	2	AGGC	RIL	-
G161	2	TCGTT	RIL	Discarded
G163	2	TGGCTA	RIL	-
G164	2	TGCTGGA	RIL	-
G168	2	TGCA	RIL	-
G169	2	AGCCC	RIL	-
G170	2	CTTCCA	RIL	-
G185	2	AATATGC	RIL	-
G202	2	GATC	RIL	-
G205	2	ACCTAA	RIL	-
G216	2	ACGTGTT	RIL	-
G218	2	AACCGAGA	RIL	-

Table S1. Continued

Samples	Plate	Barcode	Sample Type	Comments
G224	2	ACTA	RIL	-
G239	2	GTATT	RIL	-
G244	2	GAGATA	RIL	-
G248	2	ACGACTAC	RIL	-
G250	2	TCAC	RIL	-
G252	2	ATATGT	RIL	-
G255	2	ATTAATT	RIL	-
G256	2	ACAGGGAA	RIL	-
G257	2	CAGA	RIL	-
G259	2	CTGTA	RIL	-
G261	2	ATGCCT	RIL	-
G263	2	GGTGT	RIL	-
G266	2	AGGAT	RIL	-
G268	2	ATCGTA	RIL	-
G269	2	ATTGGAT	RIL	-
G270	2	ACGTGGTA	RIL	Discarded
G271	2	AACT	RIL	-
G273	2	ACCGT	RIL	-
G275	2	TATTTTT	RIL	-
G276	2	TAGCATGC	RIL	-
G277	2	ATTGA	RIL	-
G279	2	CATCGT	RIL	-
G281	2	CATAAGT	RIL	-
G284	2	CCATGGGT	RIL	-
G288	2	GCGT	RIL	-
G289	2	GTAA	RIL	-
G291	2	CTTGCTT	RIL	-
G293	2	AGTGGA	RIL	-
G294	2	CATCT	RIL	-
G295	2	CGCGGT	RIL	-
G297	2	CGCTGAT	RIL	-
G299	2	CGCGGAGA	RIL	-
G300	2	TGCGA	RIL	-
G301	2	GGTTGT	RIL	-
G302	2	ATGAAAC	RIL	-
G303	2	TAGGCCAT	RIL	-
G304	2	CCTAC	RIL	-
G305	2	CTATTA	RIL	-
G306	2	CGGTAGA	RIL	-
G307	2	CGTGTGGT	RIL	-
G308	2	CGAT	RIL	-
G311	2	CCAGCT	RIL	-

Table S1. Continued

Samples	Plate	Barcode	Sample Type	Comments
G312	2	AAAAGTT	RIL	-
G313	2	TGCAAGGA	RIL	-
G314	2	GAGGA	RIL	-
G315	2	GCCAGT	RIL	-
G317	2	CTACGGA	RIL	-
G318	2	GCTGTGGA	RIL	-
G319	2	CGCTT	RIL	-
G321	2	TTCAGA	RIL	-
G325	2	GAATTCA	RIL	-
G326	2	TGGTACGT	RIL	-
G327	2	GGAAC	RIL	-
G328	2	GGAAGA	RIL	-
G329	2	GCGGAAT	RIL	-
G330	2	GGATTGGT	RIL	-
G331	2	TCACC	RIL	-
G332	2	TAGGAA	RIL	-
G335	2	GAACTTC	RIL	-
G337	2	TCTCAGTC	RIL	-
G338	2	GTCAA	RIL	-
G340	2	GTACTT	RIL	-
G341	2	TAGCGGA	RIL	-
G342	2	GTGAGGGT	RIL	-
G343	2	CTAGC	RIL	-
G344	2	GCTCTA	RIL	-
G345	2	GGACCTA	RIL	-
G346	2	CCGGATAT	RIL	-
G347	2	TAATA	RIL	-
G348	2	GTTGAA	RIL	Discarded
G350	2	TCGAAGA	RIL	-
G351	2	TATCGGGA	RIL	-
G353	2	ACAAA	RIL	-
G355	2	CCACAA	RIL	-
G356	2	GTCGATT	RIL	-
G357	2	CGCCTTAT	RIL	-
G358	2	TACAT	RIL	Discarded
G359	2	TAACGA	RIL	-
G360	2	TCTGTGA	RIL	-
MM738	1	CTCC	P1	-
AG91-25	1	TTCTC	P2	-

[a] Water used as negative control. Less than 1,000 reads were obtained after the demultiplexing step.
[b] These RILs were excluded from the analyses presented in this paper because of insufficient number of reads or presence of contaminations.

Table S2. Mean of Temperature (T°C) and Relative Humidity (RH%) (with standard errors) measured during phenotyping assays conducted on the eggplant [MM738 × AG91-25] RIL population.

Assay name	Period of assay	Night		Day	
		T (°C)	RH (%)	T (°C)	RH (%)
TO10.Reunion	March-May 2015	21 (2)	87 (8)	32 (7)	57 (18)
TO10.Indonesia	2010	Na*	Na	Na	Na
CFBP2957-season 1	May-June 2014	18 (2)	81 (8)	29 (7)	56 (17)
CFBP2957-season 2	October-November 2014	21 (2)	86 (7)	35 (6)	49 (14)
CFBP3059-season 1	May-June 2012	18 (3)	79 (7)	23 (4)	67 (10)
CFBP3059-season 2	April-May 2013	19 (2)	90 (7)	28 (5)	66 (16)
CMR34-season 1	October-December 2012	21 (2)	87 (11)	33 (4)	58 (14)
CMR34-season 2	August-October 2013	17 (2)	95 (7)	27 (5)	56 (20)

*Not available

Table S3. Excel file with complete eggplant [MM738 × AG91-25] RIL population genetic map and significant hits on eggplant, tomato and potato genomes.

Table S4. **QTL detected for resistance to 3 strains of *R. solanacearum* species complex based on the composite interval mapping method and the Haley-Knott regression model in the [MM738 × AG91-25] RIL population.** QTL analysis for the Waudpc variable was conducted on individual seasons of PSS4, CFBP2957, CFBP3059 and CMR34 strains.

Strain	Seasons	LG[a]	Chr.[b]	QTL[c]	Location[d] (cM)	nearest marker	95% CI[e] (cM)	LOD	R^{2f}	Total R^{2g}	add effect[h]
PSS4	1	2	E02	*EBWR2*	70.6	COF324b	65.0-71.0	13.5	30.7	30.7	-7.2***
	2	2	E02	*EBWR2*	70.0	ecm009	61.0-79.0	4.1	10.5	10.5	-3.2***
CFBP2957	1	2	E02	*EBWR2*	66.0	ecm009	62.0-79.0	8.7	15.3	38.8	-6.1***
		14	E05	*EBWR14*	3.0	CDX125a	2.0-6.3	14.5	27.4		-7.3***
	2	2	E02	*EBWR2*	72.0	COI393a	64.0-74.0	16.0	28.6	43.2	-9.6***
		14	E05	*EBWR14*	3.0	CDX125a	1.0-13.2	10.2	17.0		-7.3***
CFBP3059	1	14	E05	*EBWR14*	7.8	n32451	5.0-10.2	13.8	31.8	31.8	-7.3***
	2	2	E02	*EBWR2*	66.0	ecm009	62.0-71.0	9.4	14.3	50.6	-9.1***
		14	E05	*EBWR14*	12.0	n98289	8.4-13.0	21.9	39.7		-13.6***

[a] Linkage group.
[b] Chromosome corresponding to linkage group.
[c] Name of the QTL: eggplant bacterial wilt resistance (EBWR) followed by the linkage group number.
[d] Position of the maximum logarithm of odds score (LOD) in centimorgans (cM).
[e] Bayesian confidence interval.
[f] R^2: Estimates of the proportion of phenotypic variance (percentage) explained by the QTL detected.
[g] Estimate of the total proportion of phenotypic variance explained by the additive model.
[h] Additive effect: Positive values mean that the allele comes from the MM738 parent, while negative values mean that the allele comes from the AG91-25 parent.

Table S5. Means comparison for the Waudpc variable. Eggplant RILs were grouped according to the alleles of susceptibility ("A") and resistance ("B") to bacterial wilt at *EBWR2* locus, *EBWR14* locus and on both *EBWR2* and *EBWR14* loci.

Trial	*EBWR2*[a]		*EBWR14*[b]		*EBWR2/EBWR14*			
	AA[c]	BB[c]	AA	BB	AA/AA	AA/BB	BB/AA	BB/BB
PSS4								
Sample size	76	83	-	-	-	-	-	-
Waudpc	63.2 (7.2) a[d]	52.7 (7.9) b	-	-	-	-	-	-
TO10.Indonesia								
Sample size	78	87	-	-	-	-	-	-
Waudpc	47.4 (9.0) a	31.4 (10.2) b	-	-	-	-	-	-
TO10.Reunion								
Sample size	79	90	-	-	-	-	-	-
Waudpc	80.7 (6.1) a	74.6 (8.8) b	-	-	-	-	-	-
CFBP2957								
Sample size	79	90	83	93	33	46	45	42
Waudpc	30.6 (12.7) a	16.9 (10.7) b	30.0 (13.2) a	18.0 (11.2) b	40.3 (11.0) a	23.7 (8.8) b	22.7 (9.6) b	10.4 (8.2) c
CFBP3059								
Sample size	66	81	74	81	28	38	41	38
Waudpc	32.4 (14.9) a	22.6 (13.1) b	37.6 (12.1) a	18.3 (10.0) b	46.4 (8.9) a	22.1 (8.5) b	31.8 (10.8) c	13.3 (7.7) d

[a] *EBWR2* locus is represented by the ecm009 marker.
[b] *EBWR14* locus is represented by the n32451 marker.
[c] Genotype; AA is homozygous for the P1 (MM738) susceptible allele; BB is homozygous for the P2 (AG91-25) resistant allele.
[d] Mean value, standard deviation in brackets and group assigned by the LSD test.

Table S6. List of SNPs between eggplant MM738 susceptible and AG91-25 resistant parental lines found in the candidate R gene transcripts at the *EBWR9* QTL conferring resistance to 3 phylotype-I strains of *R. solanacearum* species complex and their effects on predicted protein.

Transcript ID	SNP position[a]	MM738 genotype	AG91-25 genotype	SNP quality[b]	SNP effect on protein[c]
singlet__11792	148 (CDS)	A/A	G/G	121.91	NS (K ↔ E)
	158 (CDS)	A/A	G/G	158.72	NS (H ↔ R)
	229 (CDS)	C/C	G/G	394.42	NS (H ↔ D)
	245 (CDS)	A/A	T/T	426.42	NS (K ↔ M)
	285 (CDS)	C/C	T/T	241.49	S
	316 (CDS)	C/C	G/G	69.45	NS (Q ↔ E)
singlet__6456	174 (3’ UTR)	C/C	T/T	31.81	-
	185 (3’ UTR)	C/C	A/A	324.42	-
	193 (3’ UTR)	G/G	A/A	414.45	-
	347 (CDS)	C/C	T/T	2762.42	NS (R ↔ Q)
	374 (CDS)	C/C	T/T	2138.42	NS (G ↔ D)
	520 (CDS)	G/G	C/C	1352.42	NS (N ↔ E)
	522 (CDS)	T/T	C/C	1378.42	
	714 (CDS)	G/G	T/T	222.52	NS (Q ↔ K)
	719 (CDS)	G/G	T/T	174.62	NS (A ↔ D)
	726 (CDS)	C/C	A/A	176.62	NS (V ↔ L)
	730 (CDS)	C/C	A/A	187.62	NS (K ↔ N)
	825 (5’ UTR)	T/T	A/T	163.19	-
singlet__40855	183 (5’ UTR)	C/C	A/A	215.62	-
	257 (5’ UTR)	A/A	G/G	265.47	-
	273 (5’ UTR)	A/A	T/T	229.52	-
	372 (5’ UTR)	A/A	G/G	709.42	-
	421 (5’ UTR)	A/A	G/G	609.42	-
	503 (5’ UTR)	T/T	G/G	419.42	-
	544 (5’ UTR)	A/A	T/T	498.42	-
	768 (CDS)	C/C	T/T	125.17	NS (S ↔ F)
	947 (CDS)	T/T	G/G	158.81	NS (F ↔ V)
	1019 (CDS)	A/A	C/C	320.44	NS (N ↔ H)
	1090 (CDS)	T/T	C/C	315.44	S
	1114 (CDS)	T/T	C/C	206.62	S
	1366 (CDS)	A/A	G/G	278.47	S
	1453 (CDS)	A/A	G/G	391.42	S
	1465 (CDS)	T/T	C/C	367.43	S
	1725 (CDS)	G/G	A/A	324.44	NS (S ↔ N)
	1824 (CDS)	T/T	C/C	426.42	NS (I ↔ T)
	1916 (CDS)	A/A	G/G	244.52	NS (I ↔ V)
	1945 (CDS)	G/G	A/A	204.62	S
	2112 (CDS)	C/C	T/T	601.42	NS (S ↔ F)
	2161 (CDS)	T/T	C/C	350.43	S
	2279 (CDS)	T/T	A/A	329.44	NS (Y ↔ N)
	2290 (CDS)	A/A	C/C	285.47	S
	2791 (CDS)	C/C	A/A	308.44	S
	2841 (CDS)	T/T	C/C	323.44	NS (L ↔ P)
	2870 (CDS)	G/G	T/T	389.42	NS (G ↔ C)
	2872 (CDS)	T/T	C/C	366.43	

Table S6. Continued

Transcript ID	SNP position[a]	MM738 genotype	AG91-25 genotype	SNP quality[b]	SNP effect on protein[c]
singlet__40855	2969 (CDS)	A/A	G/G	532.42	NS (T ↔ A)
	3179 (CDS)	T/T	C/C	548.42	NS (C ↔ R)
	3496 (CDS)	T/T	A/A	662.42	S
	3649 (CDS)	C/C	T/T	579.42	S
	3661 (CDS)	G/G	A/A	645.42	S
	3755 (CDS)	G/G	A/A	259.47	NS (D ↔ N)
	3982 (3’ UTR)	G/G	T/T	323.44	-
singlet__57090	30 (CDS)	A/A	A/T	35.86	NS (N ↔ Y)
	127 (CDS)	T/T	T/C	260.19	S
	183 (CDS)	T/C	T/T	261.19	NS (S ↔ F)
	214 (CDS)	C/C	C/T	133.19	S
	271 (CDS)	A/C	C/C	397.28	NS (L ↔ F)
	361 (CDS)	T/T	T/C	228.21	S
singlet__10906	33 (CDS)	T/T	T/C	33.44	S
	48 (CDS)	A/A	A/G	74.86	S
	79 (CDS)	C/C	C/T	184.37	S
	141 (CDS)	C/A	A/T	425.19	S
	162 (CDS)	C/T	C/C	116.19	S
	179 (CDS)	G/G	A/G	79.19	NS (G ↔ E)
	225 (CDS)	C/C	C/T	237.19	S
	272 (CDS)	A/A	G/G	340.44	NS (H ↔ R)
	298 (CDS)	T/T	T/A	227.19	NS (W ↔ R)
	330 (CDS)	G/G	G/C	186.19	NS (E ↔ D)
	337 (CDS)	C/C	C/T	36.19	S
	340 (CDS)	A/G	A/A	45.44	NS (V ↔ I)
	363 (CDS)	G/G	A/G	95.19	S
	367 (CDS)	G/G	A/G	126.19	NS (E ↔ K)
	390 (CDS)	A/A	G/A	34.87	NS (I ↔ M)
	435 (CDS)	G/G	A/G	64.19	S
singlet__34133	187 (CDS)	G/G	A/A	203.62	S
	225 (CDS)	C/C	T/T	196.62	NS (A ↔ T)
singlet__13069	264 (CDS)	C/C	A/A	198.62	S
	302 (CDS)	A/A	G/G	51.85	NS (E ↔ G)
	332 (CDS)	C/C	T/T	51.85	NS (A ↔ V)
	522 (CDS)	G/G	A/A	246.47	S
	580 (CDS)	T/T	G/G	153.81	NS (S ↔ A)
	610 (CDS)	A/A	G/G	79.81	NS (I ↔ V)
	768 (CDS)	C/C	T/T	453.42	S
	1059 (CDS)	C/C	T/T	87.81	S

[a] SNP position on the transcript in bp and transcript region correspondence in parenthesis. UTR = UnTranslated Region; CDS = Coding DNA Sequence.
[b] Phred-scaled quality score for the SNP calling. High quality scores indicate high confidence calls.
[c] Effect on the SNP on the predicted protein: S = Synonymous substitution; NS = NonSynonymous substitution. When the substation is NS, the amino acid (one letter code) change is indicated in parenthesis.

2. Supplementary Figures

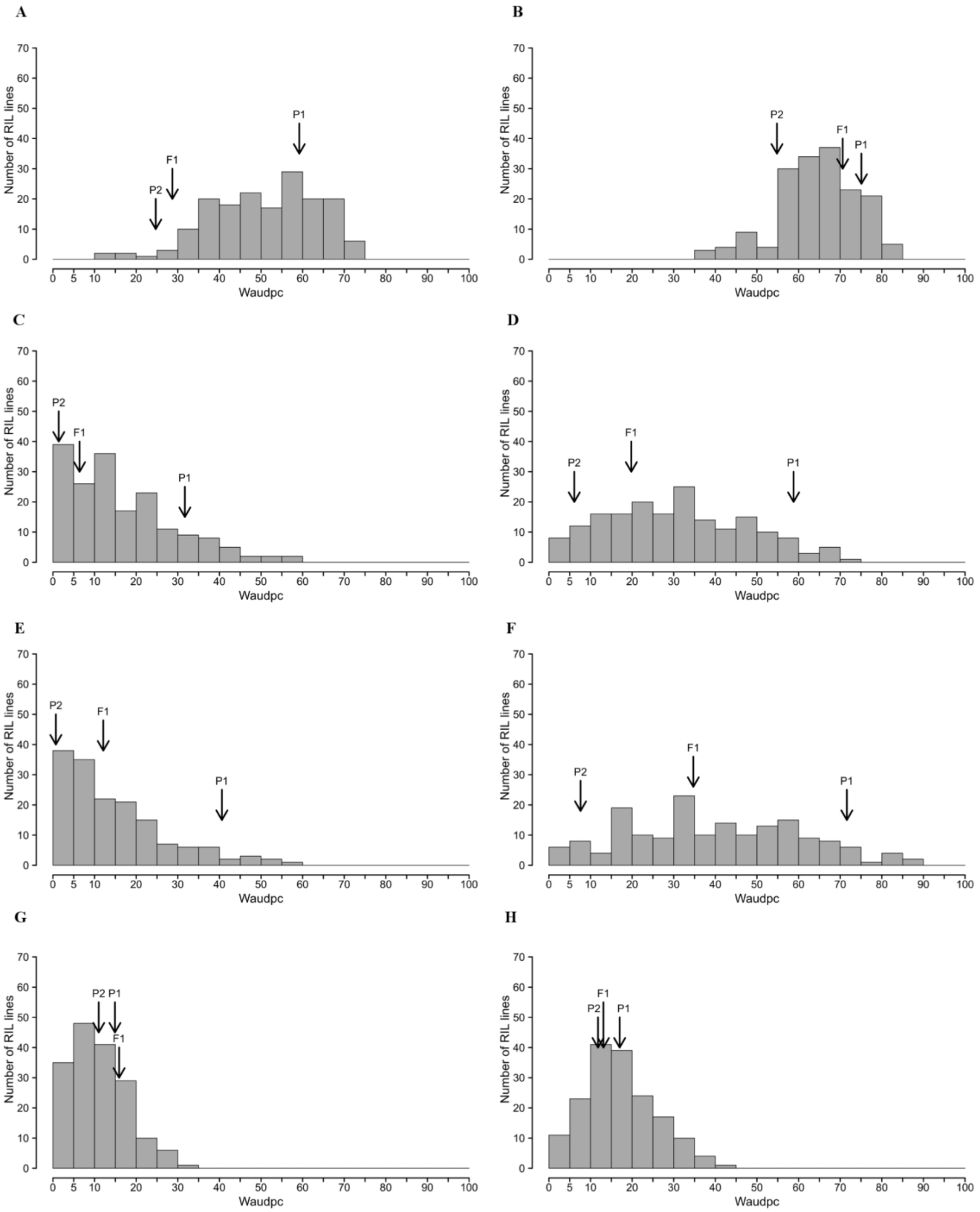

Figure S1. Frequency distribution of the Waudpc variable in the eggplant [MM738×AG91-25] RIL population obtained in individual seasons. Results are presented for: PSS4-season 1 **(A)**; PSS4-season 2 **(B)**; CFBP2957-season 1 **(C)**; CFBP2957-season 2 **(D)**; CFBP3059-season 1 **(E)**; CFBP3059-season 2 **(F)**; CMR34-season 1 **(G)** and CMR34-season 2 **(H)**. Arrows indicate the means of parental accessions MM738 (P1) and AG91-25 (P2) and of the F1 generation.

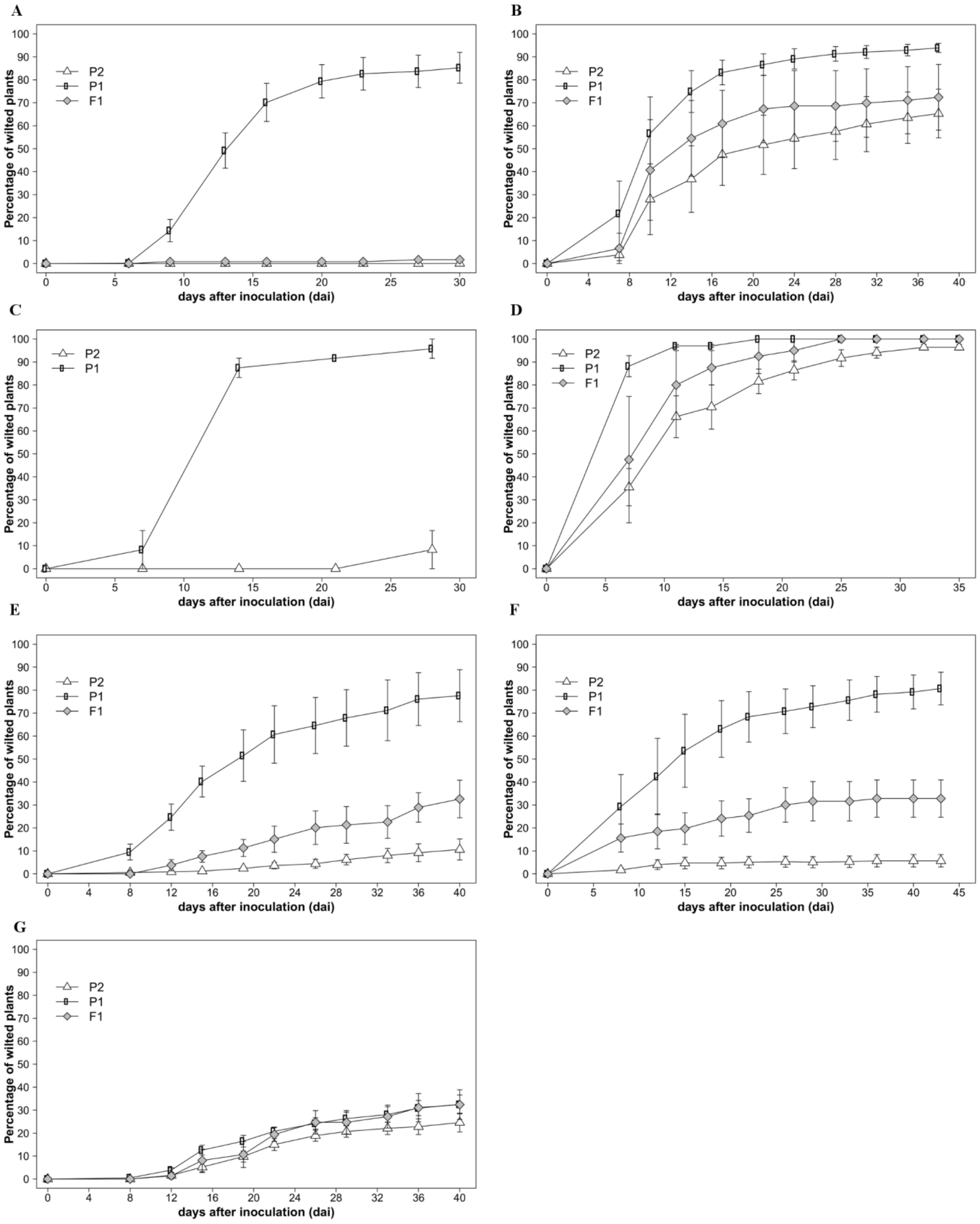

Figure S2. Bacterial wilt disease incidence progression curves for the eggplant parental lines MM738 (P1) and AG91-25 (P2) and for F1 progeny. Results are presented for the combined data of GMI1000, PSS366 and CMR134 **(A)**; for the combined seasons of PSS4 **(B)**; for TO10.Indonesia **(C)** and TO10.Reunion **(D)**; for the combined seasons of CFBP2957 **(E)**, CFBP3059 **(F)**, CMR34 **(G)**. Data for F1 progeny were not available for TO10.Indonesia.
